# Specific Roles of HSP27 S15 Phosphorylation Augmenting the Nuclear Function of HER2 to Promote Trastuzumab Resistance

**DOI:** 10.3390/cancers12061540

**Published:** 2020-06-11

**Authors:** Soo-Yeon Hwang, Seul-Ki Choi, Seung Hee Seo, Hyunji Jo, Jae-Ho Shin, Younghwa Na, Yun-Sil Lee, Youngjoo Kwon

**Affiliations:** 1 College of Pharmacy, Graduate School of Pharmaceutical Sciences, Ewha Womans University, Seoul 03760, Korea; hilarious06@naver.com (S.-Y.H.); sskchoi@naver.com (S.-K.C.); seunghee1226@naver.com (S.H.S.); angella502@naver.com (H.J.); 2 College of Pharmacy, CHA University, Pocheon 487010, Korea; wogh975@naver.com (J.-H.S.); yna7315@cha.ac.kr (Y.N.)

**Keywords:** breast cancer, trastuzumab resistance, heat shock protein 27, human epidermal growth factor receptor 2, protein-protein interaction

## Abstract

Trastuzumab (TZMB) is widely used as first line therapy for breast cancer (BC) patients overexpressing human epidermal growth factor receptor 2 (HER2). Despite its clinical benefits, many patients suffer from primary or secondary resistance to this drug within one year. As diverse molecular mechanisms occur contemporaneously during the resistance development, we focused on elucidating the role of heat shock protein 27 (HSP27) in TZMB-resistance, as this protein simultaneously regulates the function of diverse client molecules that are involved in the resistance mechanism. By extensively utilizing TZMB-refractory breast cancer cell lines transduced with diverse phosphovariants of HSP27, our study newly revealed that specific phosphorylation of HSP27 at S15 promoted its S78 phosphorylation and served as key mediator to promote direct interactions that increase the stability of HER2 and protein kinase B (AKT). This phosphorylation promoted nuclear translocation of HER2, enhancing the distinct nuclear function of HER2 that promoted AKT activation and cyclin D1 expression. Co-administration of TZMB and a functional inhibitor of HSP27, J2, significantly reduced the S15/78 phosphorylation of HSP27, which downregulated HER2 and its downstream signals, sensitizing TZMB-refractory cell, and JIMT1-xenograft mouse models to TZMB. Collectively, p-HSP27^S15^ could serve as a valuable predictive marker and also a therapeutic target for TZMB-resistance.

## 1. Introduction

Breast cancer (BC) is the most common cancer in women worldwide. According to U.S. BC statistics (breastcancer.org and the American Cancer Society), the incidence of invasive BC in U.S. women is about 12% (one in eight) over the whole lifetime. Even the risk of BC in U.S. men is increasing continuously (1 in 883 in 2019). Although BC mortality can be dramatically ameliorated by early diagnosis and treatment, invasive BC is still the malignant disease with second highest death rate (after lung cancer) [1,2].

BC is divided into four subtypes by receptor status: luminal A (estrogen receptor (ER)-positive, progesterone receptor (PR)-positive, and human epidermal growth factor receptor 2 (HER2)-negative); luminal B (ER-positive and/or PR-positive, HER2-positive); HER2 overexpressing (ER-negative, PR-negative and HER2-positive); and triple negative (ER-negative, PR-negative, HER2-negative). According to the analysis of a national cancer database (collected in collaboration with the American Cancer Society and the American College of Surgeons since 2010), 14.5% of 298,937 invasive BC cases were HER2-positive. Among them, Asian/Pacific Islanders and young women account for the highest proportion [2,3]. HER2-positivity is generally considered to predict aggressive phenotypes and poor prognosis, because HER2-amplified tumors are larger and have a higher number of positive nodes and a higher lympho-vascular invasion rate than HER2-negative tumors [2,4,5,6]. Typically, HER2 gene amplification and protein overexpression in BC are also considered to be prognostic and predictive factors for the development of metastases to the central nervous system [5,7]. 

The first line therapy for HER2-positive BC is trastuzumab (TZMB), which is the first FDA-approved human monoclonal antibody drug that targets the extracellular domain of HER2. TZMB primarily inhibits HER2 homodimerization and triggers its degradation and internalization [8,9]. TZMB is widely applied as a key component in most effective clinical regimens at this time [6,10,11]. Despite the significant clinical benefits of TZMB, its biggest limitation is primary and acquired resistance; only one third of HER2^+^ BC patients initially obtain therapeutic benefits, and many primary responders develop resistance within one year of treatment initiation [12,13,14,15,16,17]. Diverse molecular mechanisms simultaneously take place in this process, thus, the identification of critical predictive and therapeutic markers that can readily modulate various causes of TZMB-resistance at the same time remains as a major interest in both preclinical and clinical research. 

Heat-shock protein 27 (HSP27), the most abundant small heat-shock protein and redox-sensitive molecular chaperone, is ubiquitously expressed in the human body, and its expression is upregulated by oxidative stress, aging, and tumorigenesis in diverse cancers [18]. Overexpressed HSP27 in cancer is extensively related to tumor cell growth, metastasis, and the induction of various kinds of chemoresistance. HSP27 provides essential microenvironments for cancer development by stabilizing various oncogenic genes and proteins critically involved in tumor progression [19]. The association of HSP27 with BC, especially the oncogenic features and drug responsiveness of the HER2^+^ subtype, has been consistently shown in prior reports [20,21,22,23,24,25]. HSP27 extensively modulates various hallmarks of cancer progression and chemoresistance. In these processes, HSP27 is mainly regulated by phosphorylation, which induces the protein to dynamically assemble or dissociate [26]. Phosphorylation changes the affinity of HSP27 to its client oncoproteins [27] overall, in many cases leading to promotion of diverse antiapoptotic and prosurvival signaling pathways [28,29]. 

Therefore, in this study, we sought to elucidate the concrete role of HSP27 in TZMB-resistance development by clarifying how its phosphorylation is specifically regulated to facilitate the TZMB-refractoriness. Overall, our findings present a novel therapeutic approach for HER2-overexpressing BC patients that can also be effectively applied to those with TZMB-resistance.

## 2. Results

### 2.1. HSP27 Plays a Negative Prognostic Role in HER2^+^ BC Patients

**Figure 1:**
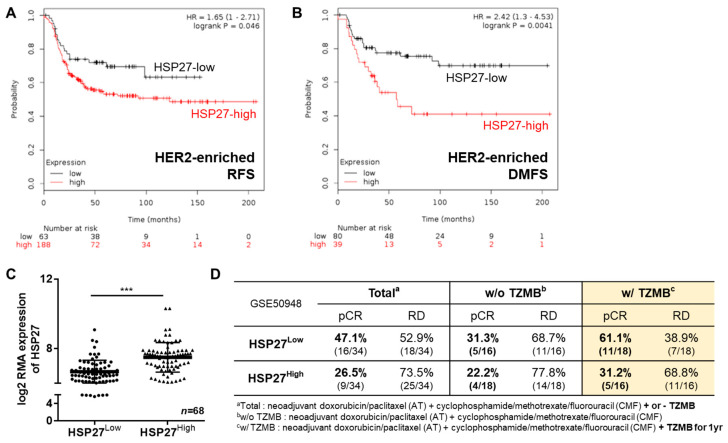
Heat shock protein 27 (HSP27) plays a negative prognostic role in human epidermal growth factor receptor 2 (HER2)-positive breast cancer (BC). (**A**) The relapse-free survival rate of patients with different HSP27 expression levels was assessed through a survival analysis using a KM plotter (http://kmplot.com/analysis). A total of 251 patients were included in the analysis. * *p* = 0.046, log rank test. (**B**) The distant metastasis–free survival probability of HSP27-high and -low patients was analyzed using KM plotter. A total of 119 patients were involved in the assessment. ** *p* = 0.0041, log rank test. (**C**) A total of 114 HER2^+^ patients from GSE50948 were classified into two groups. Those with a log2 robust multi-array average expression value of *HSPB1* in the top 30% (*n* = *34*) were assigned to the HSP27^High^ group, and those in the bottom 30% of *HSPB1* values (*n* = *34*) were categorized as the HSP27^Low^ group. Student’s t-test, *** *p* < 0.001. (**D**) The pathological complete response (pCR) and residual disease (RD) rates of the HSP27^High^ and HSP27^High^ groups were calculated. The patients in the HSP27^High^ group were more likely to remain in RD status, regardless of their regimen subtype.

Upregulation of HSP27 has previously been shown to be associated with increased tumorigenesis, metastasis, and chemoresistance in several cancer types [30,31,32,33,34,35]. Thus, to specifically verify the prognostic impact of HSP27 in HER2^+^ BC, we analyzed the survival probability of HER2^+^ BC patients using a dataset from Kaplan-Meier Plotter (KM plotter; http://kmplot.com/analysis) [36], and found that both relapse-free survival (Figure 1A; *n* = 251; HR, 1.65; 95% CI, 1–2.71; *p* = 0.046) and distant metastasis-free survival (Figure 1B; *n* = 119; HR, 2.42; 95% CI, 1.3–4.53; *p* = 0.0041) were significantly lower in patients highly expressing HSP27 than other patients. To further identify the effect of HSP27 on the chemoresistance of HER2^+^ BC, we analyzed 114 HER2^+^ BC patients from the publicly available Gene Expression Omnibus dataset (GSE50948; result from the Neoadjuvant Herceptin breast cancer trial), who received neoadjuvant doxorubicin/paclitaxel and cyclophosphamide/methotrexate/fluorouracil with or without one year of TZMB. In advance of the analysis, patients were classified into two groups by their *HSPB1* expression level; those in the top 30% of *HSPB1* expression (*n* = 34) were assigned to HSP27^High^ group, and those in the bottom 30% of *HSPB1* expression (*n* = 34) were placed in the HSP27^Low^ group (Figure 1C). Noticeably, regardless of the regimen type, patients in the HSP27^Low^ group showed distinctively higher pathological complete response (pCR) rates than the HSP27^High^ patients (HSP27^Low^ vs. HSP27^high^; 16/34 patients [47.1%] vs. 9/34 patients [26.5%]) (Figure 1D). To specifically assess the association between HSP27 and TZMB efficacy, we then compared patients who did and did not receive TZMB within the HSP27^Low^ and HSP27^high^ groups. In the HSP27^Low^ group, the pCR rate among patients who received TZMB was about 2-fold higher than among those who did not (pCR rate without TZMB vs. with TZMB: 5/15 patients [31.3%] vs. 11/18 patients [61.1%]). However, in the HSP27^High^ group, the pCR rate between the TZMB-treated and non-treated patients was similar (pCR rate without TZMB vs. with TZMB: 4/18 patients [22.2%] vs. 5/16 patients [31.2%]), and the majority remained in residual disease (RD) status (pCR vs. RD: 5/16 patients [31.2%] vs. 11/16 patients [68.8%]), indicating that an elevated HSP27 level is directly associated with reduced responsiveness to TZMB. Despite the apparent differences in the gene levels of HSP27, the *HSF1* (transcription factor of HSP27) levels in the two groups did not differ (Figure S1A), implying that elevated HSP27 was not associated with an increase in *HSF1* and *ERBB2* gene expression (Figure S1B), but instead depended on a different mechanism.

### 2.2. HSP27 is Critically Connected to TZMB-Resistance in HER2^+^ BC

**Figure 2:**
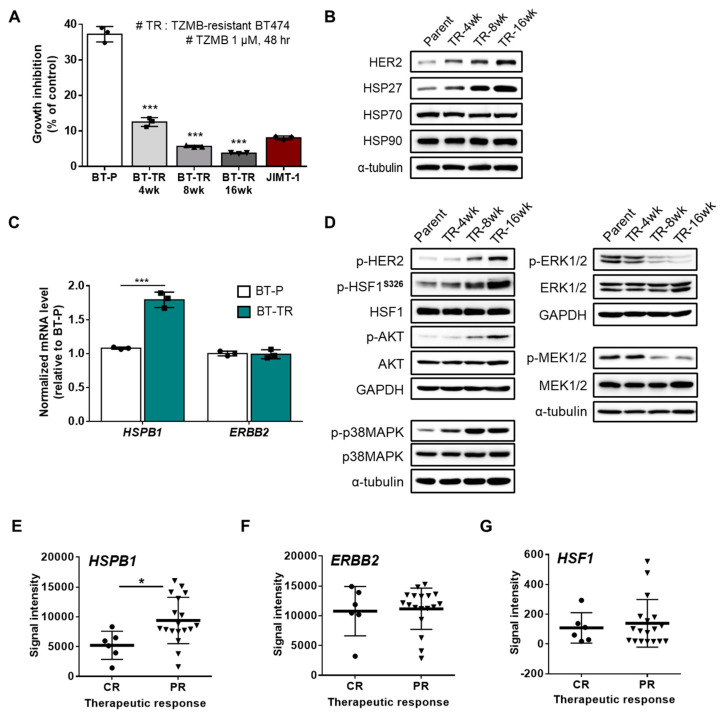
HSP27 is critically engaged in trastuzumab (TZMB)-resistance (TR) in HER2^+^ BC. (**A**) Resistance development was assessed every four weeks. The TZMB-mediated growth inhibitory effect decreased gradually and reached a level lower than that of the JIMT-1 cells at 16 weeks. Analysis of variance (ANOVA), *** *p* < 0.001 versus BT-P. (**B**) Changes in the HSP27 and HER2 levels were assessed in cell lines developing TZMB-resistance. Alterations in the HER2 level show a direct correlation with the HSP27 level. (**C**) mRNA levels of HSP27 and HER2 were evaluated in the BT-P and BT-TR cell lines. The HSP27 mRNA level was significantly upregulated in BT-TR cells, whereas no changes were observed in the HER2 mRNA level (*n = 3*). Student’s t-test, *** *p* < 0.001 versus BT-P. (**D**) Changes in several signaling pathways were comprehensively evaluated in TZMB-resistant cell lines. (**E**–**G**) Gene expression levels of HSP27 (**E**) HER2 (**F**) and HSF1 (**G**) were compared between complete responders (CR) and partial responders (PR) to TZMB (GSE62327). Significant alteration was found only in HSP27. Student’s t-test, * *p* < 0.05.

Given our finding that HSP27 is directly related to chemoresistance in HER2^+^ BC, we prepared two HER2^+^ TZMB-resistant breast cancer cell lines to elucidate the detailed role played by HSP27 in the TZMB-refractory mechanism: JIMT-1 (ER^-^/PR^-^/HER2^+^ subtype, from a TZMB-treating patient showing innate resistance) and the TZMB-refractory BT474 (BT-TR; ER^+^/PR^+^/HER2^+^ subtype; acquired resistance) (Figure S2A). To establish the BT-TR cell line, we applied TZMB to parental BT474 (BT-P, TZMB-sensitive) cells for 16 weeks, with a steady increase in the TZMB dose up to 2.1 mg/mL (14.5 μM). The growth inhibition rate of 1 μM TZMB was measured every 4 weeks to confirm successful development of resistance (Figure S2B,C). Compared with the BT-P cells, the proliferation inhibitory effect of TZMB had already decreased markedly after 4 weeks (BT-P vs. BT-TR 4-week; 37.2% vs. 12.5%; a 3.3-fold reduction), and a 10-fold reduction was finally attained at 16 weeks (BT-TR 16-week; 3.8%). The establishment process was discontinued at that point because the growth inhibition rate of the BT-TR 16-week cells against TZMB was lower than that of the JIMT-1 cells (8.1%) (Figure 2A). The acquired resistance was sustained continuously, even when TZMB was completely removed from the growth medium (data not shown).

Along with the development of resistance, cells exhibited a gradual increase in the levels of HSP27 though no noticeable changes were found in the levels of other HSPs, such as HSP70 and HSP90. Alterations in the HER2 expression level correlated directly with the HSP27 level, implying a negative association between TZMB susceptibility and both HSP27 and HER2 (Figure 2B). The upregulation of HSP27 was further found to be significantly regulated at the transcriptional level. However, the mRNA level of HER2 did not show any changes (Figure 2C), implying that the increased HER2 expression occurred mainly on the protein level. Under these conditions, the BT-TR cells exhibited significant upregulation of p-HER2 and subsequent activation of the PI3K-AKT pathway (Figure 2D), which is generally considered to be the most prominent resistance mechanism for TZMB [37]. In addition to PI3K-AKT, we found a remarkable increase in the p38MAPK pathway among the HER2 downstream signaling cascades, but a decrease in MEK1/2 and ERK1/2 activity (Figure 2D). This significant attenuation of the MEK-ERK axis is likely caused mainly by the hyperactivation of p38MAPK [38,39,40]. As p38MAPK positively and MEK/ERK negatively regulate the activation of HSF1 (increased p-HSF1^S326^)—a well-known transcription factor of *HSPB1* (gene name of HSP27) [41,42,43]—the observed changes in both the p38MAPK and MEK/ERK signaling system could have synergistically promoted the expression of *HSPB1*, increasing the total HSP27 level in the BT-TR cells overall. In addition to HER2, the upregulated HSP27 in the BT-TR cells might also have stabilized and activated various client proteins involved in the resistance mechanism, such as AKT, p38MAPK, and STAT3 [44], to continuously maintain the TZMB-refractory phenotype.

We confirmed a similar trend of significant HSPB1 upregulation in partial responders to TZMB in the publicly available GSE62327 database (Figure 2E). No significant difference was found in the ERBB2 (Figure 2F) and HSF1 (Figure 2G) gene expression levels between the complete responders and partial responders, once again supporting the clinical relevance of targeting HSP27 in TZMB-resistance.

### 2.3. HSP27 Directly Modulates HER2 and its Downstream AKT Pathway to Induce TZMB-Resistance in HER2^+^ BC

**Figure 3:**
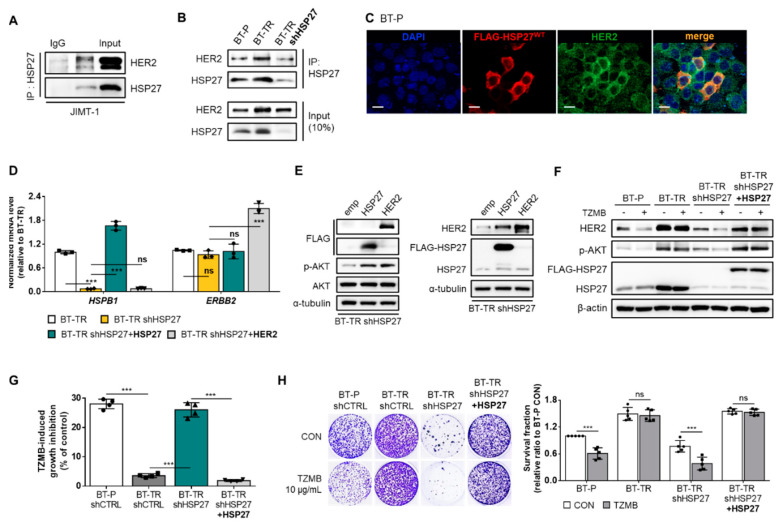
HSP27 directly modulates HER2 and its downstream protein kinase B (AKT) pathway to induce TZMB-resistance in HER2^+^ BC. (**A**) Direct interactions between HSP27 and HER2 were confirmed in JIMT-1 cells using immunoprecipitation assay. (**B**) Extent of the HSP27 and HER2 interaction was compared between BT-P and BT-TR cells. Binding between the two proteins was increased in BT-TR cells and was significantly reduced along with HSP27 knockdown. (**C**) HSP27-mediated upregulation of HER2 was confirmed by transiently overexpressing HSP27. The fluorescence intensity of HER2 was increased only in those cells in which overexpression of HSP27 was successfully induced. Scale bars = 20 μM. (**D**) Alterations in the mRNA level were assessed by silencing HSP27 in BT-TR cells and restoring HSP27 expression in BT-TR shHSP27 cells. In all cases, the mRNA level of HER2 did not change significantly (*n = 3*). ANOVA, *** *p* < 0.001. (**E**) The overexpression of HER2 and HSP27-mediated changes in the endogenous level of HER2 and HSP27 were evaluated. Transduction of HER2 did not change HSP27 expression. (**F**–**H**) Changes in TZMB responsiveness were assessed by silencing HSP27 in BT-TR cells and re-expressing HSP27 in BT-TR shHSP27 cells. HSP27 silencing significantly downregulated HER2 and phospho-AKT (p-AKT) ((**F**) 12 h treatment of TZMB), leading to short-term ((**G**) 48 h treatment of 10 μg/mL of TZMB) and long-term ((**H**) 10-day incubation with TZMB) growth inhibition in response to TZMB. Inversely, restoration of HSP27 in BT-TR shHSP27 completely rescued the TZMB-resistant phenotype. ANOVA, *** *p* < 0.001.

As previously mentioned, HER2 is a client protein of HSP27 (Figure 3A), and its stability is increased upon interaction with HSP27 [30]. Along with HSP27 overexpression in BT-TR cells, we were able to confirm that binding between HSP27 and HER2 was significantly promoted compared to that found in the BT-P cells (Figure 3B). The enhanced HSP27 and HER2 interaction in the BT-TR cells was directly reflected in the increased total level of HER2, implying that HSP27 increases the stability of HER2. In contrast, the stable silencing of HSP27 in BT-TR cells reversed the level of HER2 back to its parental state by interrupting the direct interaction between HSP27 and HER2 (Figure 3B). The transient induction of HSP27 into the BT-P cell line also directly upregulated the HER2 level, only in cells in which the overexpression of HSP27 was successfully induced (Figure 3C). However, neither HSP27 knockdown in BT-TR cells (BT-TR shHSP27) nor restoring HSP27 in BT-TR shHSP27 cells (BT-TR shHSP27 + HSP27) changed the mRNA level of HER2 (Figure 3D), indicating that HSP27-mediated HER2 upregulation occurs at the protein level, mostly by HSP27 controlling the stability of HER2 as a chaperone. In contrast, transduction of HER2 failed to affect the expression of HSP27 at the protein (Figure 3E) or mRNA levels (Figure 3D), suggesting that HER2 is insufficient to directly regulate HSP27 expression.

HSP27 knockdown in BT-TR cells not only induced a significant decrease in the endogenous HER2 level and its direct downstream AKT activation, but also allowed TZMB to induce subsequent degradation of HER2 and attenuation of the AKT pathway (Figure 3F). Rescuing the HSP27 expression (BT-TR shHSP27 + HSP27 in Figure 3F) restored the endogenous level of HER2 and p-AKT, and again inhibited the TZMB efficacy. TZMB-induced growth inhibition (Figure 3G) and long-term proliferation inhibition rate (Figure 3H) were greatly increased in the BT-TR shHSP27 cell line, reaching an extent similar to that in BT-P, but they declined back to BT-TR levels in BT-TR shHSP27 + HSP27 cells, once again confirming that HSP27 regulation could serve as an effective strategy to overcome TZMB-resistance.

### 2.4. Phosphorylation of HSP27 at Ser15 and 78 is Required for Active Interaction with HER2

**Figure 4:**
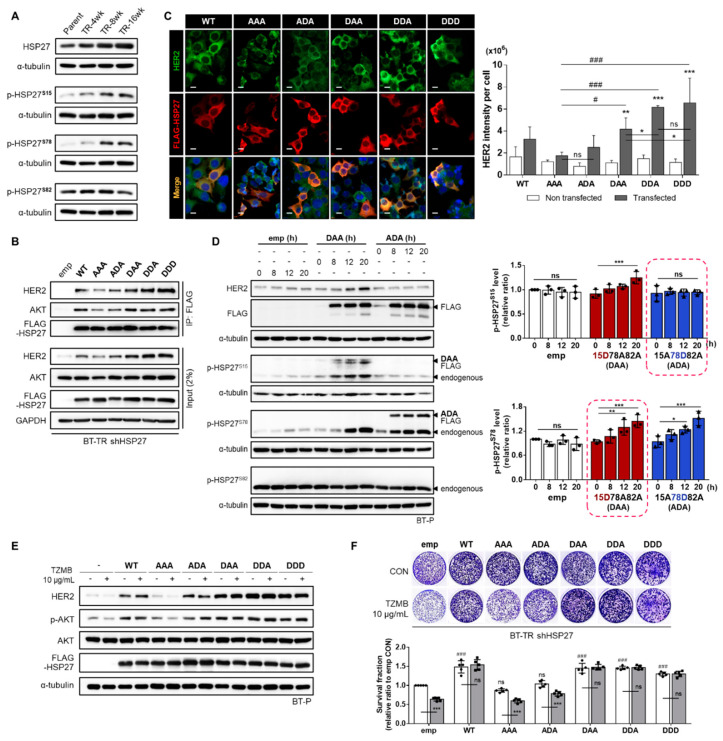
Phosphorylation of HSP27 at serine 15 and 78 is required for interaction with HER2. (**A**) Gradual changes in HSP27 phosphorylation levels were assessed in TZMB-resistant cell lines. A distinctive increase in S15 and S78 phosphorylation was observed. No changes were found in S82 phosphorylation. (**B**) Changes in the interaction between HSP27 and HER2 were examined by reconstituting BT-TR shHSP27 cells with phospho-variants of HSP27. (**C**) (Left) Changes in HER2 fluorescence intensity were evaluated by transiently overexpressing each of the indicated HSP27 phospho-variants in BT-P cells (24 h transfection). Scale bars = 20 μM. (Right) The intensity of HER2 per cell was quantified using ImageJ software (*n = 4*). Unlike the other phospho-variants, the non-phosphorylatable AAA mutant failed to make changes in the intensity of HER2. The S15D-containing mutants were confirmed to have the most significant influence on the endogenous HER2 level. ANOVA, ** *p* < 0.01, *** *p* < 0.001 vs. non-transfected cells. (**D**) Time-dependent changes in the endogenous levels of p-HSP27^S15^ and p-HSP27^S78^ were evaluated after separately and transiently transducing DAA and ADA. Pseudo-phosphorylation of S15 gradually induced the upregulation of the endogenous p-HSP27^S78^ level. ANOVA, * *p* < 0.05, ** *p* < 0.01, and *** *p* < 0.001 versus 0 h. (**E**,**F**) The effect of HSP27 phosphorylation on TZMB-sensitivity was evaluated by transiently ((**E**) TZMB was applied after 18 h of transfection and maintained for 6 h) and stably (**F**) introducing each indicated mutant to BT-P and BT-TR shHSP27 cells (14-day incubation with TZMB), respectively. The S15D-containing mutants significantly increased the stability of HER2 and prevented it from degrading in response to TZMB. ANOVA, *** *p* < 0.001 versus CON, ^###^ *p* < 0.001 versus emp CON.

In general, HSP27 phosphorylation is well-known to regulate its chaperone activity [45]. HSP27 phosphorylation induces a structural shift from large oligomers to small dimers, which promotes chaperone activity [26,46,47]. HSP27 has three available serine phosphorylation sites, S15, S78, and S82, each of which can be phosphorylated by multiple kinases. Among them, p38MAPK is generally considered to be a major mediating enzyme [48]. As demonstrated in Figure 2D, the activity of p38MAPK showed a significant increase during the development of TZMB-resistance. Therefore, we investigated changes in the p-HSP27 level (Figure 4A). In this experiment, only the p-HSP27^S15^ and p-HSP27^S78^ levels displayed a gradual increase as resistance was acquired. No evident changes were found in the p-HSP27^S82^ level. For further evaluation, we prepared five different phospho-variants along with HSP27^WT^ (WT): HSP27^S15A/S78A/S82A^ (AAA), HSP27^S15A/S78D/S82A^ (ADA), HSP27^S15D/S78A/S82A^ (DAA), HSP27^S15D/S78D/S82A^ (DDA), and HSP27^S15D/S78D/S82D^ (DDD). Each of the three sites was replaced with either a non-phosphorylatable alanine (A) or a phosphomimetic aspartate (D). We transiently and stably introduced the constructs into BT-P and BT-TR shHSP27 cells, respectively, and examined the significance of phosphorylation at each site for the HSP27-HER2 interaction and TZMB-resistance in detail.

Among the five mutants stably transduced to the BT-TR shHSP27 cell line, the non-phosphorylatable AAA mutant showed the weakest HER2 binding extent (Figure 4B). Similar trends were also found in IF results using BT-P cells transiently transfected with the mutants. AAA also failed to make significant changes in the HER2 level, confirming that HSP27 phosphorylation is required for successful HSP27-HER2 interaction (Figure 4C). Among the stably expressed mutants, DDA and DDD exhibited strongest interaction with HER2 in BT-TR shHSP27 cells. The HER2-binding degree of those two mutants was similar, implying that S82 phosphorylation might have no significant effect on HSP27-HER2 binding (Figure 4B). Another interesting point is that the interaction between the ADA mutant and HER2 was lower than that between the DAA variant and HER2 (Figure 4B,C), suggesting that HSP27 S15 phosphorylation is more critically involved in HER2 regulation than S78 phosphorylation. Unlike ADA, the S15D-containing DAA, DDA, and DDD mediated changes in the HER2 level that were clearly higher than those with the phospho-deficient AAA, though no statistically significant differences were found among the three of them (Figure 4C). At the same time, all the mutants with an S15D mutation exhibited the strongest interaction with AKT (Figure 4B), implying that S15-phosphorylated HSP27 could concomitantly perform chaperone activity for both HER2 and AKT to comprehensively consolidate the PI3K-AKT axis in the resistance mechanism.

To understand how S15 phosphorylation can independently act as a key mediator of HSP27-HER2 interaction, we used the DAA and ADA variants to investigate time-dependent changes in the endogenous levels of p-HSP27^S15^ and p-HSP27^S78^. Pseudo-phosphorylation of S15 gradually upregulated the endogenous p-HSP27^S78^ level (highlighted by the dashed box in the bottom of Figure 4D), whereas the phosphomimetic variant of S78 did not affect the endogenous p-HSP27^S15^ level (highlighted by the dashed box in the top of Figure 4D). This indicates that S15 phosphorylation primarily mediates the phosphorylation of S78 in this process. Subsequently, transient and stable induction of the S15D-containing HSP27 mutants led to significantly decreased TZMB-sensitivity in BT-P (Figure 4E) and BT-TR shHSP27 (Figure 4F) cells, respectively. Unlike the AAA variant, the DAA, DDA and DDD mutants significantly prevented HER2 from degrading in response to TZMB (Figure 4E), and also attenuated the anti-proliferative effect of the drug (Figure 4F). Corresponding activities were also found with the ADA variant, though the extent was relatively small, showing direct correlation with the preceding results that S78 phosphorylation alone is insufficient to maximize the stabilizing effect of HSP27 on HER2. These results reconfirmed that HSP27 phospho-variant-induced changes in the total HER2 expression level were not caused by alterations in the mRNA level of HER2 (Figure S3A) but rather by the stabilization of HER2. MG132 treatment generally equalized the differently controlled HER2 levels (Figure S3B).

### 2.5. Ser15-Phosphorylated HSP27 Significantly Promotes the Nuclear Localization of HER2

**Figure 5:**
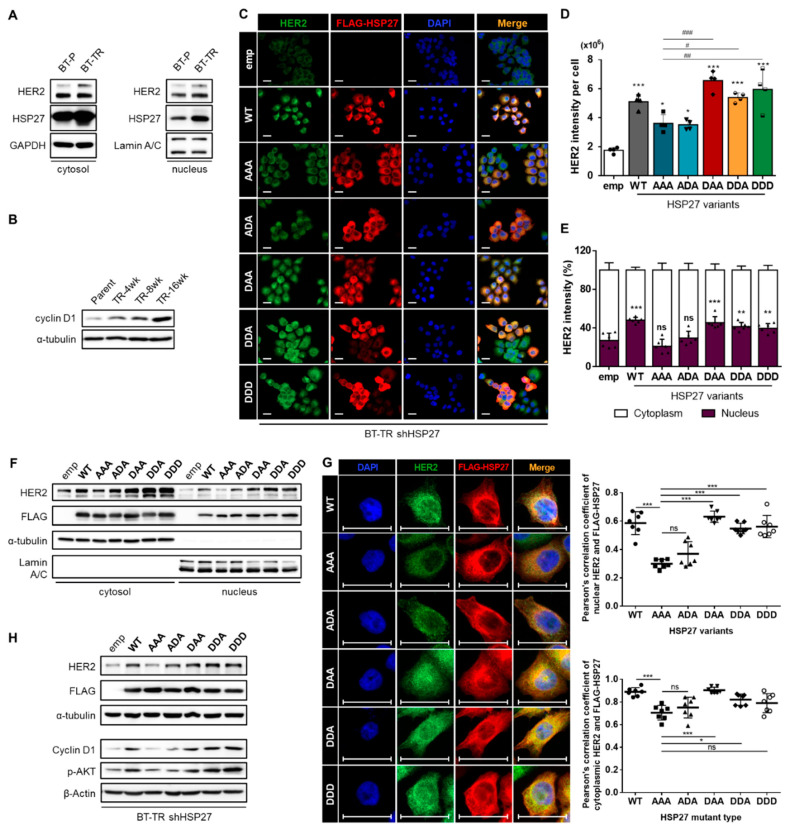
Ser15-phosphorylated HSP27 significantly promotes nuclear localization of HER2. (**A**) Alterations in the sub-cytoplasmic and sub-nuclear level of HER2 and HSP27 were evaluated separately. HER2 and HSP27 were significantly upregulated in both subcellular fractions. (**B**) Changes in the function of nuclear HER2 were assessed by examining changes in the level of cyclin D1, which is the transcriptional target of HER2. A gradual increase was observed as resistance developed. (**C**) Changes in the HER2 fluorescence intensity were evaluated in BT-TR shHSP27 cells stably reconstituted with each of the indicated HSP27 phospho-variants. Scale bars = 20 μm (**D**) Quantification of HER2 intensity per cell was conducted using ImageJ (4 images per sample). HER2 intensity was most greatly elevated when S15D-harboring mutants were introduced. ANOVA, * *p* < 0.05, *** *p* < 0.001 vs. emp, ^#^ *p* < 0.05, ^##^ *p* < 0.01, ^###^ *p* < 0.001 vs. AAA (**E**) Subcellular HER2 intensity in each of the indicated cases was quantified separately using ImageJ software. The nuclear intensity of HER2 was highest in the cases harboring S15D mutations. ANOVA, ** *p* < 0.01, *** *p* < 0.001 vs. emp. (**F**) Independent changes in the cytoplasmic and nuclear HER2 expression levels were confirmed by transiently transducing the indicated HSP27 phospho-variants to BT-P cells. Both HER2 fractions displayed significant upregulation when the S15D mutation was present (24 h transfection). (**G**) Alterations in the colocalization pattern between each HSP27 phospho-variant and HER2 were evaluated through an immunofluorescence (IF) assay. Scale bars = 20 μM. Quantification of the colocalization extent was performed using ImageJ software. (**H**) Changes in the nuclear and cytoplasmic functions of HER2 were evaluated in reconstituted BT-TR shHSP27 cells by examining alterations in the cyclin D1 and p-AKT levels.

The overexpression of HER2 and HSP27 proteins in BT-TR cells was found not only in their sub-cytoplasmic fraction, but also in the sub-nuclear compartment (Figure 5A), inducing the distinctive role of nuclear HER2 as a transcriptional coactivator that promotes cyclin D1 expression (Figure 5B). Diverse studies have previously reported that the nuclear fraction of HER2 is involved in cell growth, metastasis, invasion, and resistance to various chemotherapeutic agents in BC [49]. Particularly for TZMB, HER2 in the nucleus distinctively assembles a STAT3/HER2/HER3 nuclear complex that triggers the promoter activity of *CCND1* to escape the growth inhibitory effect of TZMB [50]. To elucidate the role of HSP27 phosphorylation in the nuclear localization of HER2, we performed an IF assay (Figure 5C). In accordance with the prior results, the average HER2 intensity per cell was higher in the groups harboring S15D (DAA, DDA, and DDD) than in the S15A-containing cases (AAA and ADA) (Figure 5C). Surprisingly, even the S15D mutation alone (DAA) was sufficient to increase the HER2 level to a similar extent to that with DDA and DDD (Figure 5D). In the case of ADA, which holds only the S78D mutation, the HER2 level was upregulated to a smaller degree than with DAA, DDA, or DDD (Figure 5D). Moreover, in accordance with the alterations in the total HER2 level, the nuclear fraction of HER2 in the AAA and ADA-expressing cells did not exhibit notable changes (Figure 5E,F). However, the phospho-variants DAA, DDA, and DDD, which commonly hold the S15D alteration, significantly upregulated the nuclear fraction of HER2 (Figure 5E,F). Along with increase in the nuclear fraction of HER2, a considerable proportion of DAA, DDA, and DDD mutants were also found in the nucleus, whereas the AAA and ADA mutants were mainly retained in the cytoplasm (Figure 5G). Strong colocalization patterns were also observed between the nuclear fraction of the DAA, DDA, and DDD mutants and HER2 (Figure 5G), suggesting that HSP27 interacts directly with HER2 not only in the cytoplasm, but also in the nucleus to enhance the distinct function of nuclear HER2, which is promoting AKT activation and cyclin D1 expression (Figure 5H).

### 2.6. J2 Attenuates the Increased HER2 Stability of TZMB-Resistant BC Cells by Inhibiting the Function of HSP27 Through Altering the Dimerization

**Figure 6:**
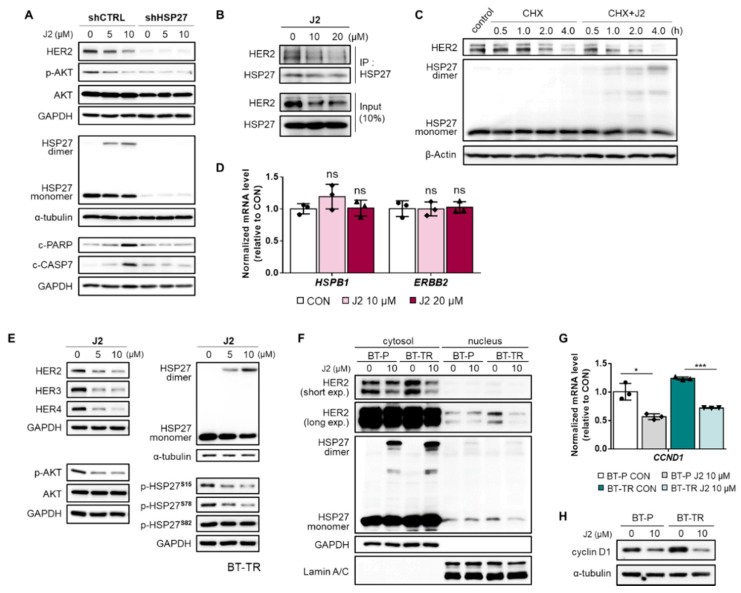
J2 attenuates HER2 stability in TZMB-resistant BC cells by inhibiting the function of HSP27 through altering dimerization. (**A**) The activity of J2 as a direct functional inhibitor of HSP27 was confirmed using BT-TR shHSP27 cells. The compound lost its activity to create abnormal dimers of HSP27 and failed to downregulate HER2 and its downstream AKT pathway (12 h treatment at indicated concentrations). (**B**) The effect of J2 on the HSP27–HER2 interaction was evaluated by co-IP assay. The interaction between HER2 and HSP27 was significantly reduced in a dose-dependent manner by treatment with J2 (12 h treatment at the indicated concentrations). (**C**) J2-mediated downregulation of HER2 stability was assessed using a cycloheximide (CHX) chase assay. Co-treatment with CHX (5 μg/mL) and J2 (20 μM) accelerated the degradation of HER2 by creating altered dimers of HSP27. (**D**) Changes in the mRNA level of HSP27 and HER2 were measured after applying the compound at the indicated concentrations. No significant alterations were found in either HSP27 or HER2 mRNA expression (12 h treatment at indicated doses, qRT-PCR, *ACTIN* as loading control). (**E**) The effect of J2 on HER-related signaling molecules was evaluated in BT-TR cells by applying the compound in the indicated concentrations. Significant, concentration-dependent attenuation was observed in HER-family proteins and the downstream AKT pathway (12 h treatment at indicated doses). (**F**) Changes in the HER2 level in different subcellular fractions of BT-P and BT-TR cells were examined, to evaluate the influence of J2 on the cytoplasmic and nuclear functions of HER2. A significant decrease was observed in both fractions of HER2 (12 h treatment). (**G**,**H**) Consequential downregulation of cyclin D1 mRNA (G, 12 h treatment, *ACTIN* as loading control) and protein ((**H**) 12 h treatment) was observed following J2 treatment. ANOVA, * *p* < 0.05, *** *p* < 0.001 versus CON.

Based on the results demonstrating the significance of HSP27 to the TZMB-resistance, we applied a functional inhibitor of HSP27, J2, and further investigated the therapeutic relevance of targeting HSP27 to overcome TZMB-resistance. This compound has previously been shown to form abnormal dimers of HSP27 that inhibit its chaperone activity by creating covalent bonds with the cysteine thiol group in HSP27 [51]. Treating BT-TR cells with J2 significantly reduced the HER2 level and its downstream AKT pathway in a concentration-dependent manner by inducing altered dimerization of HSP27 (Figure 6A). J2 completely lost its action when HSP27 was stably silenced, confirming that the HER2 downregulating effect of this compound depends directly on its HSP27 inhibitory activity. J2-induced downregulation of HER2 was facilitated by reducing the interaction between HSP27 and HER2 (Figure 6B), which eventually led to decreased HER2 protein stability (Figure 6C). However, this compound had no effect on the mRNA level of either HER2 or HSP27 (Figure 6D), indicating that all the demonstrated actions of J2 on HER2 depend solely on its inhibition of the chaperone function of HSP27.

In addition to HER2, J2 also mediated the significant attenuation of other HER-family proteins, HER3 and HER4. These proteins are generally responsible for forming heterodimers with HER2 to activate alternative signaling cascades and counteract the inhibitory action of TZMB [18] (Figure 6E, Figure S4A). Specifically, HER3 is the key heterodimerization partner of HER2 in the nucleus, and is mainly responsible for activating AKT pathway and inducing TZMB-resistance [37]. Thus, J2-mediated inhibition of HER3 and the downstream AKT signaling cascade demonstrates its important role in attenuating the distinctive nuclear function of HER2. In fact, HER2 downregulation induced by J2 affected both the cytoplasmic fraction of HER2 and the nuclear fraction (Figure 6F), leading to a remarkable decrease in the gene transcription of cyclin D1 (Figure 6G) and its overall protein expression level (Figure 6H). The same trends were found in JIMT-1 cells (Figure S4B–D), indicating that this compound can control HSP27-mediated HER2 regulating activity in any TZMB-resistant cell line that primarily overexpresses HER2. Moreover, J2 specifically downregulated S15 and S78 phosphorylation of HSP27 (Figure 6E), additionally contributing to the dissociation of HSP27 and HER2 (Figure 6B). However, J2 had no direct effect on the kinase activities of various signaling molecules within the HER2-related pathways, including MAPKAPK-2, which is the most prominent kinase to phosphorylate HSP27 (Figure S4E). Once again, the various J2-mediated regulatory effects on HER2 and its downstream signaling cascades are shown to be induced by HSP27 inhibition.

### 2.7. J2 Sensitizes TZMB-Resistant Cell Lines and Successfully Restores Significant Anticancer Activity of TZMB

**Figure 7:**
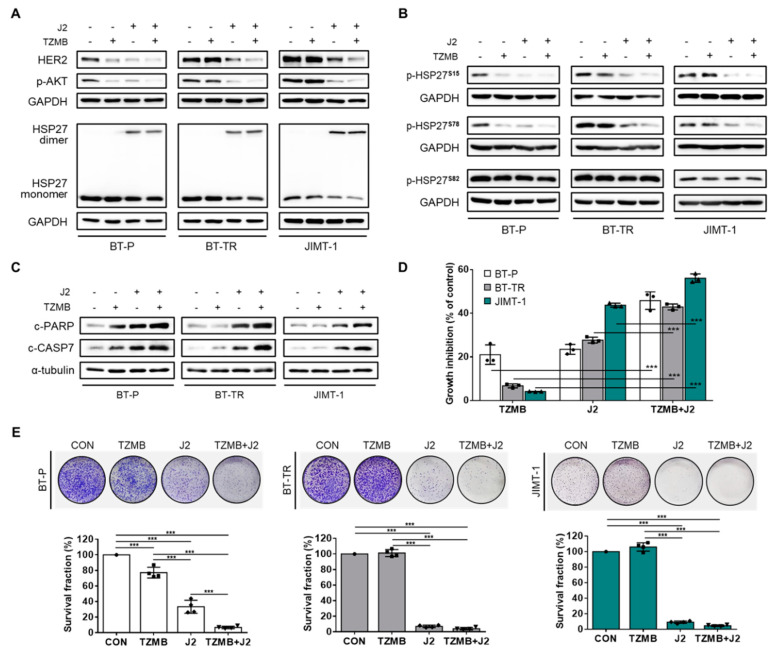
J2 sensitizes TZMB-resistant cell lines to successfully restore significant anticancer activity of TZMB. (**A**) The effect of co-treatment with J2 (10 μM) and TZMB (10 μg/mL) on HER2 and its downstream AKT signaling pathway was assessed in three different cell lines, BT-P, BT-TR, and JIMT-1. The HER2 and p-AKT levels were most significantly reduced in the co-treatment group, which also showed enhanced alterations in HSP27 dimerization (12 h treatment of both drugs). (**B**) Marked reduction in S15 and 78 phosphorylation was observed in the co-treatment group. No changes were found in S8 phosphorylation. The experimental conditions used in (**A**) were also applied here. (**C**) The effect of co-treatment with J2 (10 μM) and TZMB (10 μg/mL) on apoptosis signaling was assessed in three different cell lines, BT-P, BT-TR, and JIMT-1. Pro-apoptotic markers in the co-treatment group were significantly upregulated, along with enhanced alterations in HSP27 dimerization (24 h treatment of both drugs) (**D**) Remarkable synergism of growth inhibitory effect was observed in the group co-treated with J2 and TZMB (48 h treatment of both drugs). ANOVA, *** *p* < 0.001 versus TZMB-only group. (**E**) Long-term proliferation rate was also markedly inhibited in the TZMB + J2 group (10-day incubation). ANOVA, *** *p* < 0.001 versus CON.

To further investigate the therapeutic relevance of HSP27 inhibition as a strategy for overcoming TZMB-resistance, we assessed the anticancer effect of a TZMB+J2 combination treatment on BT-TR and JIMT-1 cells using BT-P cells as a comparative control. TZMB single treatment downregulated HER2 and its downstream AKT pathway in BT-P, but not in the TZMB-resistant BT-TR and JIMT-1 cell lines (Figure 7A). As in the preceding results, J2 alone mitigated the downregulation of HER2 and p-AKT, but the effect was maximized when the compound was co-administered with TZMB (Figure 7A). This event specifically involved distinctive downregulation of S15 and S78 phosphorylation, but no changes were observed in the level of p-HSP27^S82^ (Figure 7B). TZMB activity in the refractory cell lines is offset by the phosphorylation-mediated protective effect of HSP27 on HER2. However, when TZMB was co-administered with J2, phosphorylation of both S15 and S78 was greatly attenuated, along with an increased in the abnormal dimerization of HSP27. The synergistic effect of TZMB and J2 was subsequently followed by the significant induction of apoptosis (Figure 7C) and short-term (Figure 7D) and long-term anti-proliferation (Figure 7E) effects in TZMB-resistant cell lines. Therefore, concomitant administration of J2 and TZMB can sensitize refractory cell lines to TZMB, producing to remarkable anticancer effects. The same trends were confirmed in an in vivo xenograft mouse model of JIMT-1.

**Figure 8:**
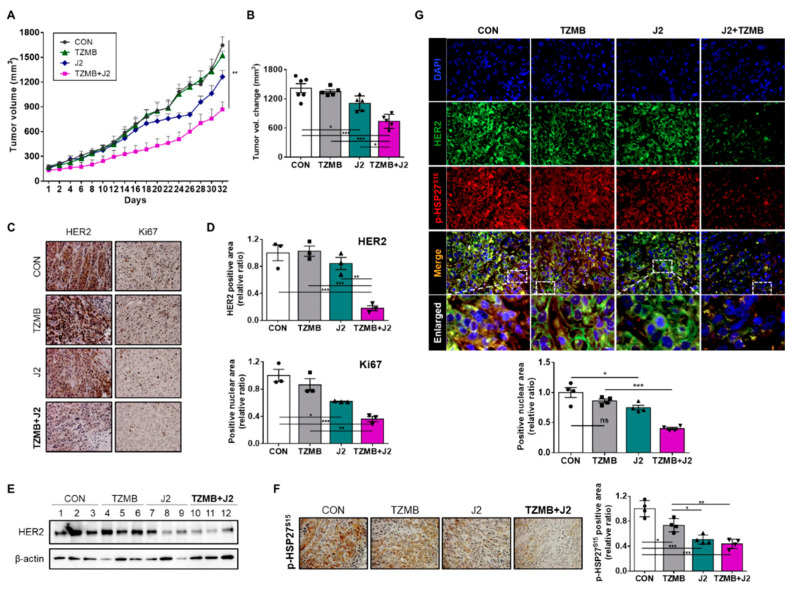
J2 successfully restored anticancer activity of TZMB in JIMT-1 xenograft mice. (**A**) In vivo tumor growth retardation effect of J2 was assessed using JIMT-1 xenograft mouse model (*n*=6 per group, intraperitoneal injection, J2 was administered every other day, and TZMB was applied once a week). Tumor volumes were calculated by measuring the length and width of the tumor with calipers and using the equation (length x width^2^)/2 (mean ± S.E.M). Student’s t-test, * *p* < 0.05 versus control. (**B**) The tumor volumes of the mice from each treatment groups were compared with control tumors, all of which were excised immediately after sacrificing the mice (*n* = 6 per group). Student’s t-test, *p < 0.05 versus CON. (**C**) Representative IHC images of HER2 and Ki67 from the tumors just described. 20x magnification. (**D**) The HER2 and Ki67 IHC scores were calculated using ImageJ software. * *p* < 0.05, ** *p* < 0.01, and *** *p* < 0.001 versus CON. (**E**) HER2 expression levels of the tumors excised from each of the groups. (**F**) Representative p-HSP27^S15^ IHC images of tumors from each of the groups. 200x magnification. Quantification was performed using ImageJ software. * *p* < 0.05, ** *p* < 0.01, and *** *p* < 0.001 versus CON. (**G**) Representative IF images of HER2 and p-HSP27^S15^ in tumor tissue from the JIMT-1 xenograft mice treated with each of the indicated drugs. Quantification was made on the merged nuclear intensity of HER2 and p-HSP27^S15^ normalized by DAPI intensity and conducted in ImageJ (four images per sample). 400x magnification. ANOVA, ns = non-significant, * *p* < 0.05, ** *p* < 0.01, *** *p* < 0.001.

The tumor growth rate was most retarded in the group that received both TZMB and J2 (Figure 8A), and the final tumor volume change was smallest in that group (Figure 8B). Our IHC analysis also revealed that HER2 level was most remarkably downregulated in the tumor tissues of the co-treated group (Figure 8C–E), along with a significant decrease in the Ki67 level (Figure 8C,D), which indicates a reduced proliferation rate. Moreover, the phosphorylation level of HSP27 S15 and the nuclear translocation of HER2 and p-HSP27^S15^ were greatly reduced in tumor tissues excised from the TZMB + J2 group (Figure 8F,G), once again confirming the finding that downregulating the chaperone activity of HSP27 by inhibiting S15 phosphorylation could allow TZMB-refractory BC patients to obtain clinical benefit from TZMB, by reducing the stability of overexpressed HER2, and placing it in a pregnable state.

## 3. Discussion

HER2 overexpression is found in 15–20% of BC patients worldwide, and is well-known to significantly reduce the disease-free and overall survival in this patient group [52]. For this reason, TZMB maintains a high clinical market share despite readily occurring resistance [3]. Various TZMB-resistance mechanisms have been demonstrated, generally involving diverse escape mechanisms that compensate the anticancer activity of TZMB. They mostly involve genetic and epigenetic alterations, inducing the constitutive reactivation of HER2 signaling, especially the PI3K/AKT/mTOR axis [53]. Despite the large amount of preclinical and retrospective evidence supporting the previous theories, their clinical relevance remains uncertain, because each of independent mechanism occurs randomly due to tumor heterogeneity, which precludes the simultaneous treatment of all resistant clones. Therefore, novel strategies are needed to comprehensively treat various refractory mechanisms at the same time. In this context, we focused on the role of HSP27 in TZMB-resistance, because it is a well-known chaperone molecule that regulates the functions of diverse clients, and many previously reported target proteins of HSP27 [43]—including HER2, the primary target of TZMB,—are involved in TZMB-resistance.

In general, the function of HSP27 is context-specifically regulated by phosphorylation in different pathological states [54]. In this study, we found that along with gradual increase in p38MAPK, the S15 and S78 phosphorylation were specifically upregulated during the TZMB-resistance development. Particularly, S15 phosphorylation primarily promoted the phosphorylation of S78 in this process, and essentially mediated the interaction between HSP27 and HER2 both in the cytoplasm and nucleus (Figure 4A–D and Figure 5A), consolidating the activation of AKT signaling pathway. HSP27 phosphorylation generally induces dissociation of large oligomers into dimers that are small enough to passively translocate into the nucleus [55]. Many recent studies have suggested that these dimeric HSP27 are the active, substrate-binding form [43,44,45,56,57]. Thus, our new finding that HSP27-mediated upregulation of the HER2 nuclear function (Figure 5A,B) can be interpreted as an event that were primarily assisted by HSP27 S15 phosphorylation, leading to increased colocalization of p-HSP27^S15^ and HER2 within the nucleus (Figure 5D–H). It is very encouraging to find that p-HSP27^S15^ could promote the non-canonical nuclear activity of HER2 as a transcriptional regulator, because HER2 in the nucleus accelerates the proliferation of BC cells more strongly than membrane HER2, allowing cells to readily escape from TZMB activity and metastasize to distant organs [34]. The upregulated nuclear HER2 level in our resistant BT-TR cell line, which finally led to increase in cyclin D1 expression (Figure 5B), apparently enhanced its basal proliferation rate compared with the parental cell line, BT-P (Figure 3H), indicating that the resistant cell line exhibited more aggressive phenotypes than its parent cell line.

Under this condition, we observed that downstream signaling cascades of HER2, such as the PI3K/AKT, MEK/ERK, and p38MAPK pathways interactively regulated one another to promote the transcriptional activity of HSF1 (Figure 2D,E), finally inducing the overexpression of HSP27. The activation of PI3K/AKT and p38MAPK signaling generally promotes the activating phosphorylation of HSF1 at S326 [41,42,43] and suppresses the inhibitory phosphorylation at S307 by significantly attenuating the MEK/ERK pathway [58]. Inhibited MEK/ERK signaling can potentially induce the activation of AKT [59], intensifying the PI3K/AKT pathway. Thus, as an inevitable event promoted by complex crosstalk among the signaling pathways, overexpression of HSP27 accompanied by specific S15 phosphorylation can be interpreted as an adaptive change to create and maintain the intracellular conditions to be favorable for bypassing the activity of TZMB.

As shown by our excellent in vitro (Figure 7C–E) and in vivo (Figure 8A,B) results, treating TZMB-resistant cells with J2 comprehensively inhibited all the resistance mechanisms described in this study. By creating abnormal, inactive dimers of HSP27 (Figure 6A), J2 significantly downregulated the phosphorylation of HSP27 at S15 and S78 (Figure 6E), eventually promoting the degradation of both the cytoplasmic and nuclear HER2 in response to TZMB treatment by lowering their protein stability (Figure 6F). It is encouraging that J2 also significantly downregulated HER3, which is a critical heterodimerization partner required by HER2 to engage in its nuclear activity as a transcriptional regulator (Figure 6E). Noticeably, J2 displayed even better anticancer activity in the BT-TR cell line than in BT-P as HSP27, the target of J2, was highly upregulated in BT-TR cells (Figure 7E). Nevertheless, J2 demonstrated the best anticancer activity when it was co-administered with TZMB (Figure 7D,E and Figure 8A,B). J2 acted as a sensitizer to TZMB by significantly downregulating HER2 stability both in the cytoplasm and nucleus, making it vulnerable to TZMB.

Moreover, J2 exhibited outstanding anticancer activity also in the BT-P cell line in vitro and in vivo. Administering J2 together with TZMB, significantly improved the survival rate of mice with BT-P xenografts (Figure S5A,B), indicating that the functional inhibition of HSP27 can demonstrate prominent anticancer activity in any cases where HSP27 is upregulated. Taken together, the patients with high level of endogenous HSP27 expression or high level of p-HSP27^S15^ could be classified as a risk for aggravated tumor conditions such as metastasis and chemoresistance. For the HER2^+^ BC patient group, the level of HSP27 expression or p-HSP27^S15^ should be continuously monitored, even if their initial drug responses are promising, and co-administration of HSP27 inhibitors should be considered. Therefore, our results provide valuable insights that can be used to develop new strategies for various HER2^+^ BC patients, including TZMB-resistant patients.

## 4. Materials and Methods

### 4.1. Expression Profile Datasets

All publicly available gene expression datasets used for therapeutic response rate analysis, GSE50948 and GSE62327, were obtained from the National Center for Biotechnology Information (NCBI) gene expression omnibus database (GEO).

### 4.2. Cells and Cell Culture

BT474 breast cancer cells were cultured in RPMI-1640 medium (Welgene Inc., Gyeongsan-si, Gyeongsangbuk-do, Korea) supplemented with 5% fetal bovine serum (FBS) (Corning Inc., Corning, NY, USA) and 1% penicillin and streptomycin (HyClone Lab Inc., Logan, UT, USA). JIMT-1, a trastuzumab-resistant breast cancer cell line, was grown in Dulbecco’s Modified Eagle Medium (Welgene), supplemented with 5% FBS (HyClone) and 1% penicillin and streptomycin (HyClone). All media were changed every 2–3 days, and the split ratios were from 1:4 to 1:10 according to the ATCC^®^ descriptions. All cells were cultured in an incubator at 37 °C with 5% CO_2_.

### 4.3. Transient Transfection of Various Plasmid Contructs

For transient transfection of plasmids, BT474 parent cells or BT-TR shHSP27 cells were plated and incubated for 24 h to reach 70% confluency. Cells were then transfected with the designated plasmids in each experiment using jetPRIME transfection reagent (Polyplus transfection, Illkirch, France) according to the manufacturers protocol. All plasmids utilized in the study are as follows: p3xflag-myc-HER2, p3xflag-myc-HSP27**^WT^**, p3xflag-myc-HSP27**^S15A/S78A/S82A^**, p3xflag-myc-HSP27**^S15D/S78A/S82A^**, p3xflag-myc-HSP27**^S15A/S78D/S82A^**, p3xflag-myc-HSP27**^S15D/S78D/S82A^**, and p3xflag-myc-HSP27**^S15D/S78D/S82D^**

### 4.4. Stable Cell Line Generation

For the generation of the HSP27 knockdown BT-TR stable cell line (BT-TR shHSP27), we first prepared BT-TR cells in 6 well plate (SPL, Pocheon-si, Gyeonggi-do, Korea) and incubated until it reached 50%–60% confluency. Cells were then infected with lentiviral particles obtained from Santa Cruz Biotechnology (Santa Cruz, CA, USA), according to the manufacturer’s protocol. For the establishment of each BT-TR shCTRL and BT-TR shHSP27 cell lines, control shRNA lentiviral particle (sc-108080), HSP27 shRNA lentiviral particle (sc-29350) was utilized, respectively. To select the stable clones expressing the designated shRNA, 4 μg/mL of puromycin (Sigma Aldrich, St. Louis, MO, USA) were applied.

To additionally generate reconstituted BT-TR shHSP27 cell lines that stably express wild-type HSP27 or different HSP27 phospho-variants (AAA, ADA, DAA, DDA and DDD), cells were prepared in a 6-well plate (SPL, Pocheon-si, Gyeonggi-do, Korea), and transfected with each of the indicated plasmids using jetPRIME transfection reagent (Polyplus transfection, Illkirch, France) as mentioned above. A total of 2 mg/mL of G418 (Biomax, Seoul, Korea) was used for the final selection of the clones that stably express each of the five phosphovariants.

### 4.5. Cell Viability Assay

Cells were cultured in 100-mm cell culture dishes until they showed 80–90% confluency. Cells were trypsinized with trypsin-EDTA and seeded at 10^4^ cells/well in a 96-well cell culture plate for an additional 20 hours. The existing medium was removed from each well and replaced with 100 µL of the designated medium without FBS or penicillin-streptomycin in an incubator at 37 °C and 5% CO_2_ for 4 h. After 4 h of starvation, compounds diluted in medium were added at the designated concentrations and left for indicated time period at each of the figures. After incubation, each well received 5 µL of EZ-cytoX (DoGenBio, Seoul, Korea). The results were finally detected after 4 h incubation at 37 °, using a M200 microplate reader (Tecan, Männedorf, Switzerland) at 450 nm.

### 4.6. Co-immunoprecipitation (Co-IP) Assay

TZMB-resistant BT474 and JIMT-1 cells were seeded in 100-mm cell culture dishes and cultured until they reached 70–80% confluency, and then the indicated compounds (5 μM) were added for 16 h. A cell lysate preparation was added, and a protein quantification method was performed using the same procedure as described below for the Western blot analyses. A total of 700 μg of cell extracts were incubated with 2 μg of control mouse IgG (Cell Signaling Technology, Danvers, MA, USA), or each of the target protein primary antibodies, at 4 °C overnight on a rotator. Then, 20 μL of protein-A/G PLUS agarose beads (Santa Cruz, CA, USA) were added to each sample and incubated for an additional 4 h at 4 °C. The agarose beads were washed 3 times with 200 μL of lysis buffer (RIPA). After removing the supernatant, the beads were eluted with 2X sample buffer by boiling at 98 °C for 5 min. The immunoprecipitated proteins were loaded and separated by SDS-PAGE and analyzed using Western blot analyses.

### 4.7. Western Blot Analysis

Cells were lysed in RIPA buffer consisting of 50 mM Tris, 0.25% sodium deoxycholate, 0.1% SDS, 150 mM NaCl, 1% NP-40, 1 mM EDTA and 1% protease inhibitor cocktail (GenDEPOT, Katy, TX, USA). Equal amounts of proteins (20–30 μg) were subjected to SDS-PAGE and transferred to a 0.2 μm PVDF membrane (Pall Life Science, Port Washington, NY, USA). Membranes were blocked with 5% skim milk or 5% bovine serum albumin (LPS solution, Daejeon, Korea), and then incubated with primary antibodies at 4 °C overnight. The blots were washed with tris-buffered saline (TBS)-0.1% Tween20 and incubated with horseradish-peroxidase-conjugated secondary antibodies. Bands were visualized using ECL solution reagent (GE Healthcare Life Sciences, Little Chalfont, UK) and LAS-3000 (Fuji Photo Film Co., Ltd., Tokyo, Japan). Images were analyzed with Multi-Gauge Software (Fuji Photo Film Co. Ltd.). The antibodies used in these experiments are listed in Table S1.

### 4.8. Quantitative Real-Time PCR

FavorPrep™ Tri-RNA reagent (FAVORGEN Biotech Corp., Taiwan) and a PrimeScript™ RT reagent kit (Takara Bio Inc., Tokyo, Japan) were used according to the manufacturers’ instructions to extract RNA from cells and synthesize complementary DNA (cDNA), respectively. Quantitative analysis of the demonstrated genes was performed using a SensiFAST™ SYBR No-ROX kit (Bioline Reagent, Ltd., London, UK). PCR amplification was conducted using a CFX96TM real-time PCR detection system (Bio-Rad, Hercules, CA, USA) under the following protocol: polymerase activation at 95 °C for 2 min, followed by 30 cycles of amplifications at 95 °C for 10 sec, 56 °C for 10 sec, and 72 °C for 20 sec. The relative quantity of mRNA was determined using the ∆∆Ct method, and normalized to *GAPDH* or *ACTIN*. The primer sequences used in this study are listed in Table S2**.**

### 4.9. Clonogenic Assay

Cells were plated in 6-well culture plates at a density of 2000 cells/well. Compounds were applied to the cells immediately after seeding. After 10 days or 14 days of incubation with or without various compounds (10 μg/mL TZMB, 10 μM J2), cells were fixed with 100% methanol for 1 h and stained with 200 μL of crystal violet solution (1% [w/v] in absolute methanol) per well. Cells were rinsed with tap water and analyzed. The images were taken using ChemiDoc bio-image analyzer (Bio-Rad, Hercules, CA, USA) and quantified by ImageJ software (NIH, Bethesda, MD, USA). All steps after fixation were performed at room temperature.

### 4.10. Kinase Inhibition Assay

The kinase inhibitory activity of J2 at 10 µM and 25 µM was evaluated by Millipore Kinase Profiling Services. Its inhibitory effects toward c-RAF, EGFR, ErbB2, ErbB4, PI3 kinases, PKB, MAPKAPK-2, and MAPKAPK-3 were analyzed according to the Kinase Profiler Service assay protocols [60]. The scintillation values were calculated as the percent of kinase inhibition with respect to the control.

### 4.11. Immunofluorescence (IF) Assay in Various Cell Lines

Cells were plated in 8-well chamber slides (SPL, Pocheon-si, Gyeonggi-do, Korea) in advance. When the cells reached 80% confluency, they were washed with phosphate-buffered saline (PBS) and fixed with 4% paraformaldehyde for 20 min at room temperature. Cells were then washed twice with PBS. After blocking with a blocking solution consisting of 5% blocking one-P (Nacalai Tesque, Kyoto, Japan) and 0.1% triton-X in PBS, cells were washed again twice with PBS. The primary antibody in dilution buffer (blocking solution diluted 2 times with PBS) was applied to the cells and incubated overnight at 4 °C. After washing the cells with PBS three times, the secondary antibody in dilution buffer was added and incubated for 1 h at room temperature. A total of 0.1 μg/mL of DAPI was added to the samples and left for 10 min to stain the nuclei. Cells were finally washed again three times, treated with Dako mounting solution (Agilent, Santa Clara, CA, USA), and sealed with cover glass. Images were taken using an apotome laser-scanning microscope and analyzed with Zen pro software (Carl Zeiss Co. Ltd., Jena, Germany). The antibodies used in these experiments are listed in Table S1.

### 4.12. Cycloheximide Assay

Cells were plated in a 6-well plate (SPL, Pocheon-si, Gyeonggi-do, Korea) and incubated for 24 h. The cells were then treated with 5 μg/mL of cycloheximide alone, or in combination with 20 μM J2. Right before the compound treatment, the growth medium of the cells was completely replaced with serum-free medium. After the compound-treated cells were incubated for the indicated times, they were harvested by trypsinization, and washed once with PBS. The collected cells were kept at −20 °C until the last sample harvest and then all of them lysed with RIPA to obtain whole protein extracts. The analysis of the protein degradation rate of HER2 and HSP27 was made using Western blots. Β-Actin was used as the loading control.

### 4.13. Subcellular Fractionation

The cytoplasmic and nuclear fractions of all cells were collected using NE-PER^®^ Nuclear and Cytoplasmic Extraction reagents (Thermo Scientific, Waltham, MA, USA). Sample preparation was made in accordance with protocol provided by the manufacturer. The final protein concentration of each sample was measured through a BCA protein assay kit (Thermo Scientific). Equal amounts of protein were applied for a Western blot analysis to evaluate changes in the subcellular localization of each protein of interest. Lamin A/C was used as the loading control for the nuclear fraction, and GAPDH was used for the cytoplasmic fraction.

### 4.14. In Vivo Xenograft Mouse Models

A single JIMT cell suspension (1 × 10^7^ cells) was injected subcutaneously into the hind legs of 4-week-old female NOD-SCID (Koatech, Pyeongtaek, Gyeonggi-do, Korea) mice, and BT474 cells (1 × 10^7^ cells) were injected into 5-week-old BALB/c nude mice (Orientbio, Seongnam, Gyeonggi-do, Korea). When the tumors reached a minimal volume of 100–200 mm^3^, the mice were treated once every 2 days with **J2** (20 mg/kg) and once a week with trastuzumab (1 mg/kg) by i.p. injection. Tumor volumes were determined according to the formula (L × W^2^)/2, by measuring tumor length (L) and width (W) with a caliper. Tumors were measured twice weekly and allowed to grow for 5 weeks.

### 4.15. Immunohistochemistry (IHC) and IF Assays in Tissue Sections

IHC was performed with the following antibodies: p-HSP27^S15^ (79-181, Prosci) or HER2 (2242S, Cell Signaling Technology) and mouse anti-Ki-67 (M7248, Dako, CA, USA). For antigen retrieval, slides were placed in citric acid buffer (pH 6.0) and heated at 100 °C for 10 min. Slides were incubated overnight at 4 °C with antibodies. Slides were then incubated with avidin-biotin peroxidase complex (ABC kit, Vector Laboratories, CA, USA) and developed using 3, 3′-diaminobenzidine tetrachloride (Zymed Laboratories, South San Francisco, CA, USA). Images were quantified using ImageJ software. All statistical analyses of the images were conducted in GraphPad Prism software 5.0 (GraphPad Software, Inc., La Jolla, CA, USA). For IF staining, tissue sections stained with HER2 and p-HSP27^S15^ (1:100 dilution; 2242S, Cell Signaling Technology, 79–181, Prosci, respectively) were incubated with appropriate fluorescent secondary antibodies and counterstained with DAPI. Images were viewed under a confocal microscope (LSM700, Zeiss, Jena, Germany).

### 4.16. Statistical Analyses

All experiments were performed at least triplicate, and the mean ± standard deviation is presented for all data. Statistics were calculated by one-way analysis of variance (ANOVA) or student’s t-test with Prism 6.0 (GraphPad Software, Inc., La Jolla, CA, USA), and the differences between two values were considered to be statistically significant when the *p* values (described using single, double and triple asterisks, respectively) were < 0.05, < 0.01, and < 0.001.

## 5. Conclusions

**Figure 9:**
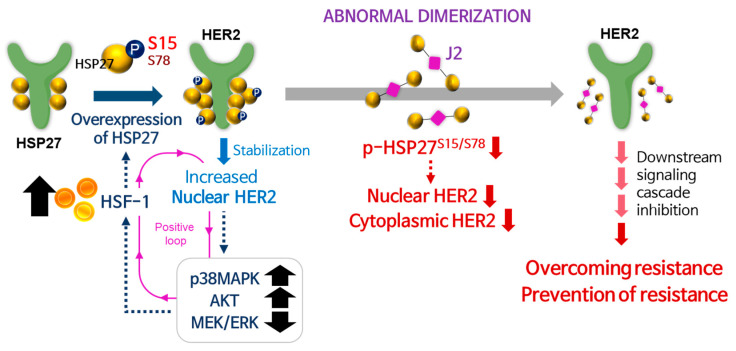
Graphical summary of overall findings of the study. HSF1-mediated HSP27 upregulation and its S15 phosphorylation leads to increase in HER2 nuclear function, reducing the TZMB susceptibility overall. Administrating J2, a functional inhibitor of HSP27, can serve as an effective overcoming and/or preventing strategy for TZMB-resistance.

Increased HSP27 chaperone activity through augmented Ser15 phosphorylation could induce TZMB-resistance by promoting HER2 stability, HER2 nuclear translocation, AKT/p38MAPK signaling, and HSF1 activation. Therefore, p-HSP27^S15^ could serve as a valuable predictive marker and also a therapeutic target for TZMB-resistance. Novel functional HSP27 inhibitor, J2 effectively sensitized TZMB-resistant BCs to TZMB by specifically downregulating the S15/S78 phosphorylation of HSP27. This overall promoted the degradation of cytoplasmic/nuclear HER2, and attenuated the downstream signaling cascade. Overall impact of this study is graphically summarized in Figure 9.

## Supplementary Materials

The following are available online at https://www.mdpi.com/2072-6694/12/6/1540/s1, Figure S1: HSP27 plays a negative prognostic role in HER2-positive BC. Figure S2: HSP27 is critically engaged in TZMB-resistance in HER2+ BC. Figure S3: Phosphorylation of HSP27 at ser15 and 78 is required for active interaction with HER2. Figure S4: J2 attenuates HER2 stability in TZMB-resistant BC cells by inhibiting HSP27 functioning through altered dimerization. Figure S5: J2 maximizes the efficacy of TZMB in breast cancers overexpressing HSP27. Table S1: General information of antibodies utilized in the study. Table S2: Information of utilized PCR primers in this study.

## Abbreviations

BC: Breast cancer; ER: Estrogen receptor; HER: Human epidermal growth factor receptor; HSP27: Heat shock protein 27; HSP70: Heat shock protein 70; HSP90: Heat shock protein 90; HSF1: Heat shock factor 1; PI3K: phosphatidylinositol 3-kinase; AKT: protein kinase B; p38MAPK: p38 mitogen activated protein kinase; IF: Immunofluorescence; IHC: Immunohistochemical staining; IP: Immunoprecipitation; PR: Progesterone receptor; qRT-PCR: quantitative real-time PCR; TZMB: Trastuzumab; TR: Trastuzumab-resistance.
