# Supplementary material for: Specific Roles of HSP27 S15 Phosphorylation Augmenting the Nuclear Function of HER2 to Promote Trastuzumab Resistance
Source: Cancers (Basel). 2020 Jun 11;12(6):1540. doi: 10.3390/cancers12061540 (PMC7352409; doi:10.3390/cancers12061540)
Supplement: Supplementary file 1 [file cancers-12-01540-s001.pdf]

cancers

Supplementary Materials: Specific Roles of HSP27 S15 Phosphorylation Augmenting the Nuclear Function of HER2 to Promote Trastuzumab Resistance

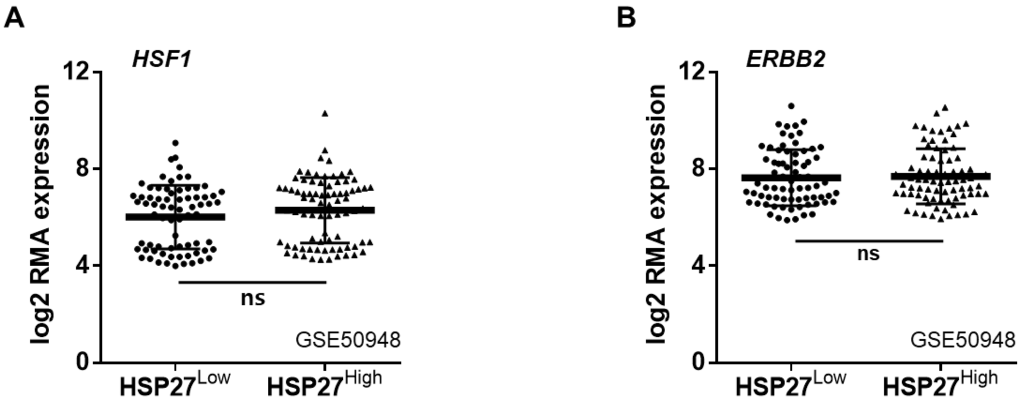

Figure S1. HSP27 plays a negative prognostic role in HER2-positive BC. A, B**.** The log2 robust multi-array average expression values of HSF1 and ERBB2 were compared between the assigned HSP27^{Low} (n = 34) and HSP27^{High} (n = 34) groups. No significant differences were found between the groups in HSF1 or ERBB2. Ns = non-significant.

A *Characteristics of selected breast cancer cells*

	p53	HER2	ER	PR	HSP27
BT474	Mu	+++	+	+/-	+++
JIMT-1	Mu	++++	-	-	++

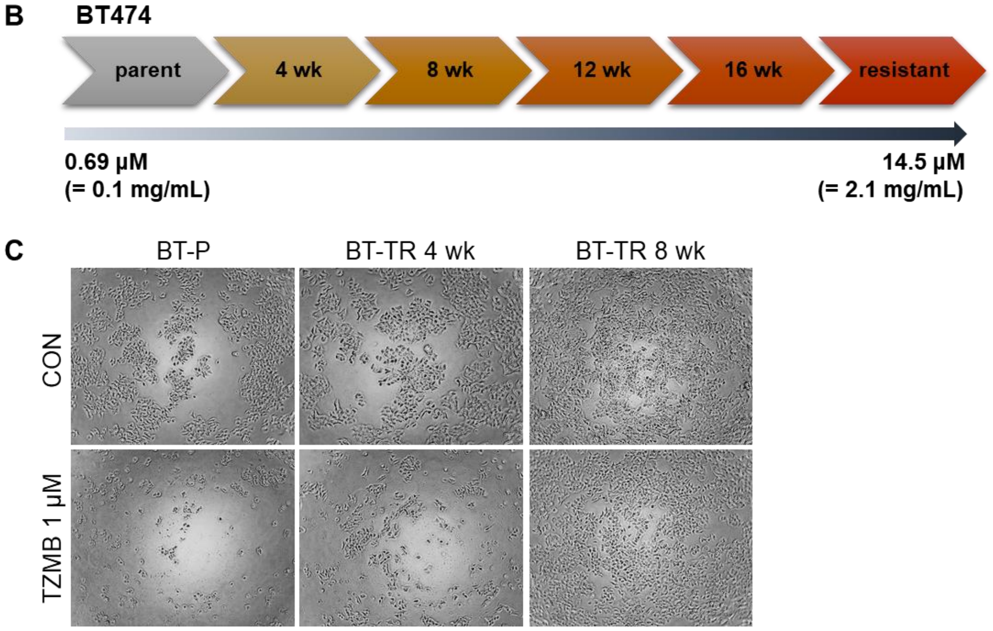

Figure S2. HSP27 is critically engaged in TZMB-resistance in HER2⁺ BC. **A.** The molecular characteristics of the cell lines used in this study are summarized. **B.** General scheme used to establish the BT-TR cell line. TZMB was applied to BT-P cells for 16 weeks with a gradual increase in the TZMB dose up to 2.1 mg/mL. TZMB was discontinued after 16 weeks. **C.** Resistance development was assessed every 4 weeks. Representative images.

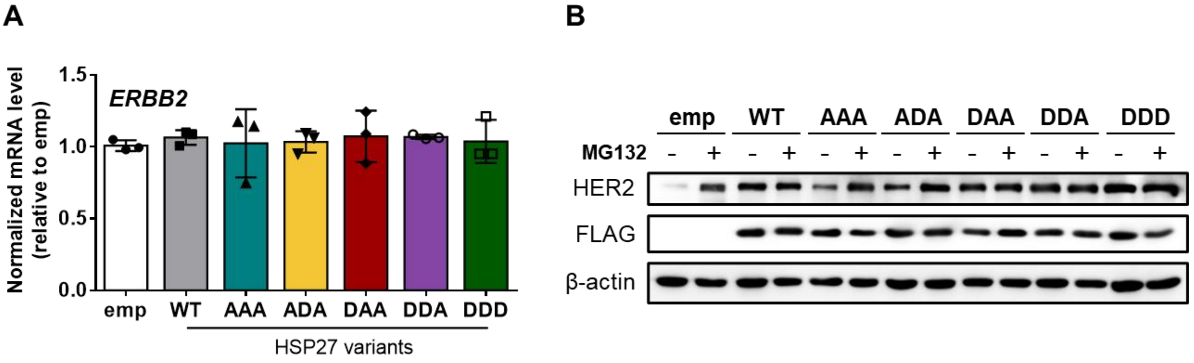

Figure S3. Phosphorylation of HSP27 at ser15 and 78 is required for active interaction with HER2**. A.** Endogenous mRNA levels of HER2 were measured in reconstituted BT-TR shHSP27 cells with each of the phospho-variants of HSP27. No significant changes were induced by the transduction of the HSP27 phospho-mutants. **B.** Changes in the HER2 expression level in reconstituted BT-TR shHSP27 cells were examined along with MG132 treatment (20 μM, 8 h treatment). Treatment with MG132 equalized the differently controlled HER2 levels.

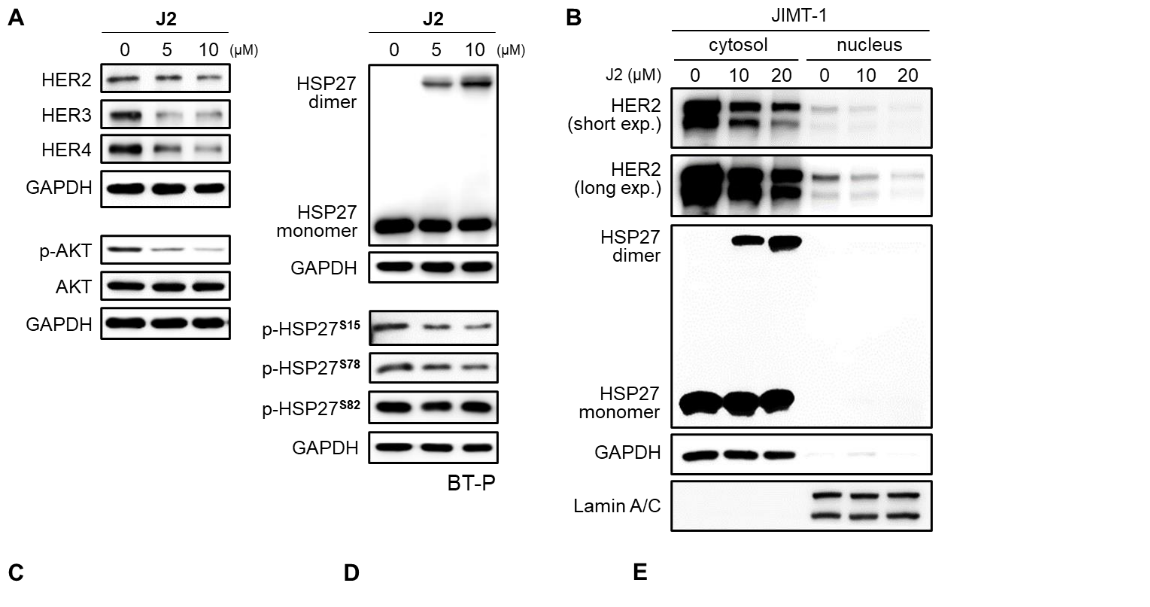

Kinase activity	J2 (10 μM)	J2 (20 μM)
EGFR (h)	101	93
ErbB2 (h)	93	92
c-RAF (h)	104	101
PI3 Kinase (h)	101	101
PKBα (h)	104	118
MAPKAPK-2 (h)	106	105
MAPKAPK-3 (h)	104	102

Figure S4. J2 attenuates HER2 stability in TZMB-resistant BC cells by inhibiting HSP27 functioning through altered dimerization. A. The effect of J2 on HER-related signaling molecules was evaluated

in BT-P cells. Significant attenuation was observed in HER-family proteins and the downstream AKT pathway (12 h treatment at indicated doses). B. Changes in the level of HER2 in different subcellular fractions of JIMT-1 cells were examined to evaluate the influence of J2 on the cytoplasmic and nuclear functions of HER2. Significant decreases were observed in both HER2 fractions (12 h treatment). C,D. Consequential downregulation of cyclin D1 mRNA (C, 12 h treatment, *ACTIN* as loading control) and protein (D, 12 h treatment) levels were observed with J2 treatment. ANOVA, *** p<0.001 versus CON. E. The direct *in vitro* kinase inhibitory activity of J2 over several HER-related kinases was measured. All the values are shown in terms of mean activity, which indicates the kinase activity remaining in the presence of the target compound. J2 had no direct effect on the HER2-related kinases.

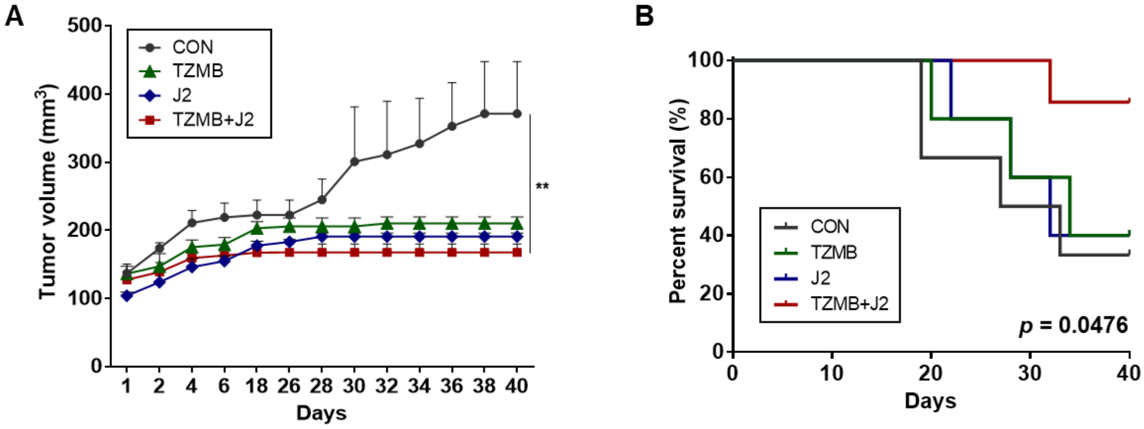

Figure S5. J2 maximizes the efficacy of TZMB in breast cancers overexpressing HSP27. **A.** *In vivo* tumor growth retardation effect of J2 was assessed using a BT-P xenograft mouse model. Only the data points of survived mice were used to generate the graph of tumor volume progression (n=3 for CON, TZMB and J2 group, n=6 for TZMB+**J2** group, intraperitoneal injection, J2 was administered every other day, and TZMB was applied once a week). **B.** Survival analysis was performed on the xenograft mouse models implanted with HSP27-highly expressing BT-P to assess the efficacy of TZMB + J2. Administering **J2** together with TZMB, significantly improved the survival rate of mice with BT-P xenografts.

Table S1. General information of antibodies utilized in the study.

	Antibody	Company	Catalog number	Dilution	Application
primary	AKT	Cell signaling	#9272	1:2000	WB
	c-PARP	Cell signaling	#9541	1:2000	WB
	HSP27	Santa cruz	sc-13132	1:2000<br>2 μg/sample	WB<br>IP
	GAPDH	MBL	M171-3	1:10000	WB
	HER3	Cell signaling	#4754s	1:1000	WB
	HER4	Cell signaling	#4795s	1:1000	WB
	HER2	Thermo	MA5-13105	1:1000<br>1:100	WB<br>IHC
	HER2	Cell signaling	#2165s	1:2000<br>1:100	WB<br>IF
	MAPK	Cell signaling	#9102	1:2000	WB
	FLAG	MBL	M185-3L	1:2000<br>2 μg/sample	WB<br>IP
	FLAG-Cy3	Sigma-Aldrich	A9594	1:250	IF
	p-AKT (S473)	Santa cruz	sc-7985	1:2000	WB
	p-MAPK (T202/Y204)	Cell signaling	#9101	1:2000	WB
	α-tubulin	Cell signaling	#2144	1:2000	WB

	**β-actin**	**Cell signaling**	**#4967**	**1:2000**	**WB**
	**Cyclin D1**	**Cell signaling**	**#2922**	**1:2000**	**WB**
	**Caspase7**	**Cell signaling**	**#9492**	**1:1000**	**WB**
	**Bcl-2**	**Santa cruz**	**sc-7382**	**1:1000**	**WB**
	**HSF-1**	**Santa cruz**	**sc-17757**	**1:1000**	**WB**
	**p-HSF-1 (S326)**	**Abcam**	**ab76076**	**1:2000**	**WB**
	**p-HSP27 (S15)**	**Cell signaling**	**#2404s**	**1:1000**	**WB**
	**p-HSP27 (S78)**	**Cell signaling**	**#2401s**	**1:1000**	**WB**
	**p-HSP27 (S82)**	**Cell signaling**	**#2405s**	**1:1000**	**WB**
**secondary**	Anti-mouse-HRP	Genetex	GTX213111-01	1:5000	WB
	Anti-rabbit-HRP	Genetex	GTX213110-01	1:5000	WB
	Anti-rabbit-Alexa fluor 488	Cell signaling	#4408s	1:100	IF

Table S2. Information of utilized PCR primers in this study.

Genes		Sequence
Actin	Forward	5' AGCCATGTACGTAGCCATCC 3'
	Reverse	5' CTCTCAGCTGTGGTGGTGAA 3'
ERBB2	Forward	5' GGTGGTCTTTGGGATCCTCA 3'
	Reverse	5' ACCTTCACCTTCCTCAGCTC 3'
GAPDH	Forward	5' GAGTCAACGGATTTGGTCGT 3'
	Reverse	5' GACAAGCTTCCCGTTCTCAG
HSPB1	Forward	5' CCTGGATGTCAACAACTTCG 3'
	Reverse	5' CTGGGATGGTGATCTCGTTG 3'
CCND1	Forward	5' CCTAAGTTCGGTTCCGATGA 3'
	Reverse	5' ACGTCAGCCTCCACACTCTT 3'

Related to Figure 2B

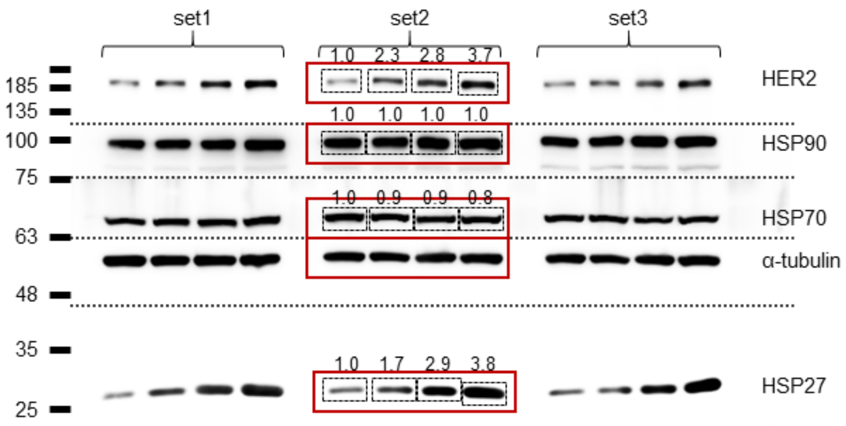

Related to Figure 2D

Black # : normalized by loading control
Purple # : normalized by total protein (phospho-protein/total protein)

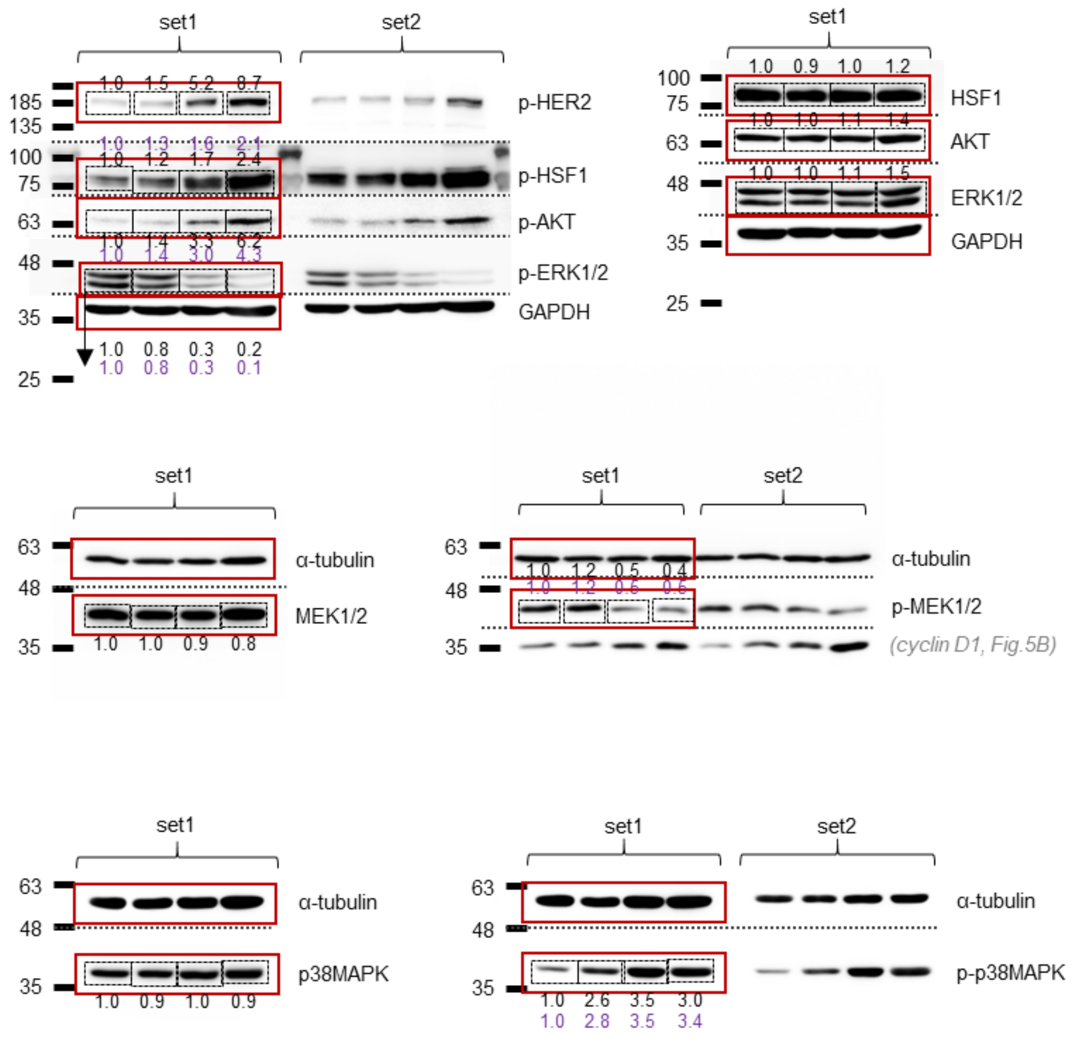

Related to Figure 3A

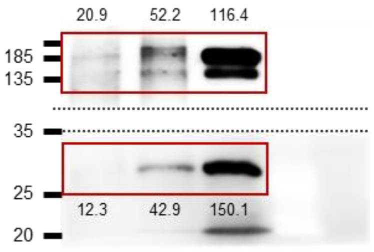

Related to Figure 3B

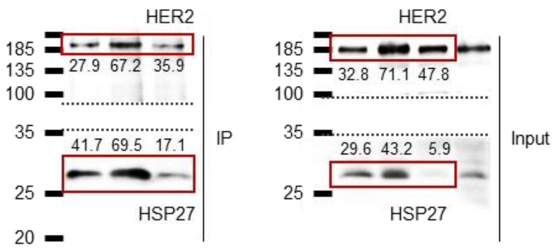

Related to Figure 3E

Black # : normalized by loading control

Purple # : normalized by total protein (phospho-protein/total protein)

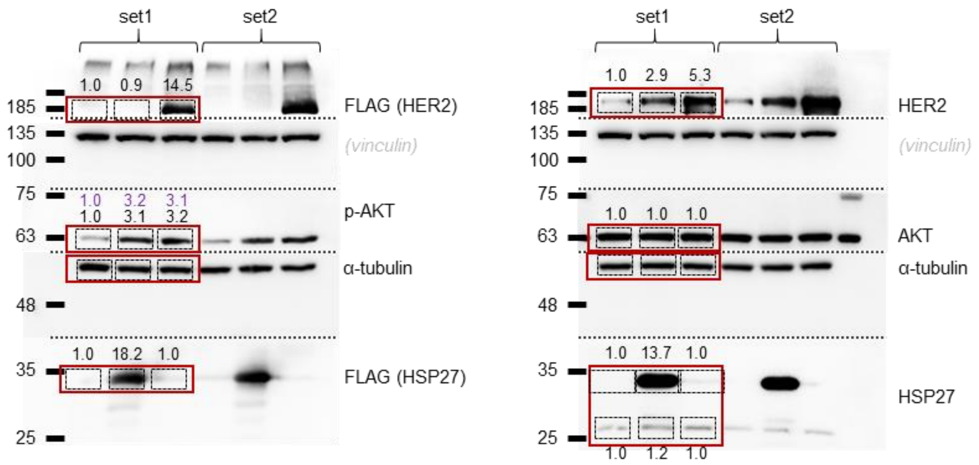

Related to Figure 3F

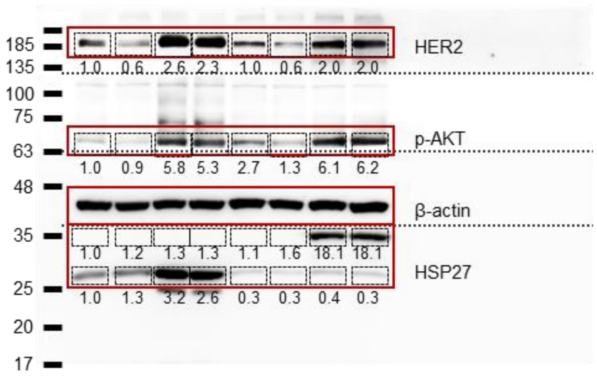

Related to Figure 4A

Black # : normalized by loading control
Purple # : normalized by total protein (phospho-protein/total protein)

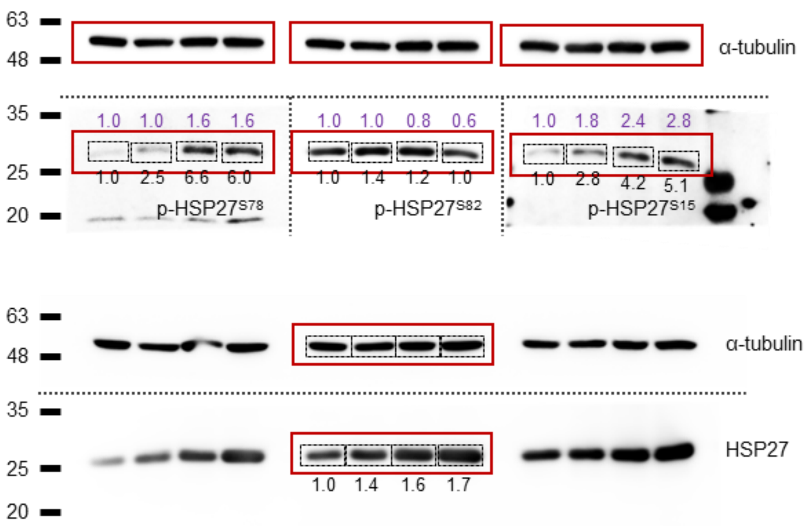

Related to Figure 4B

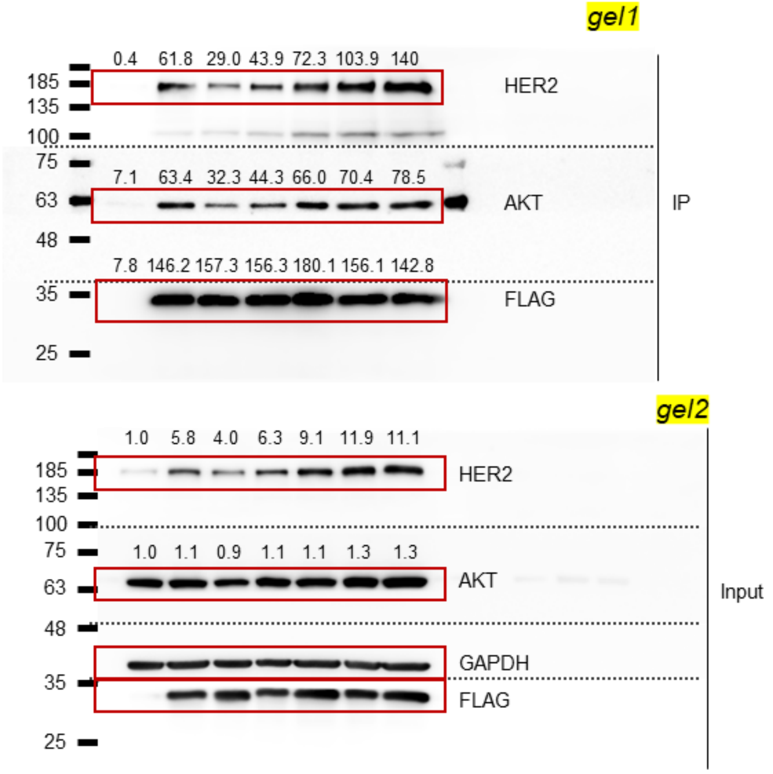

Related to Figure 4D

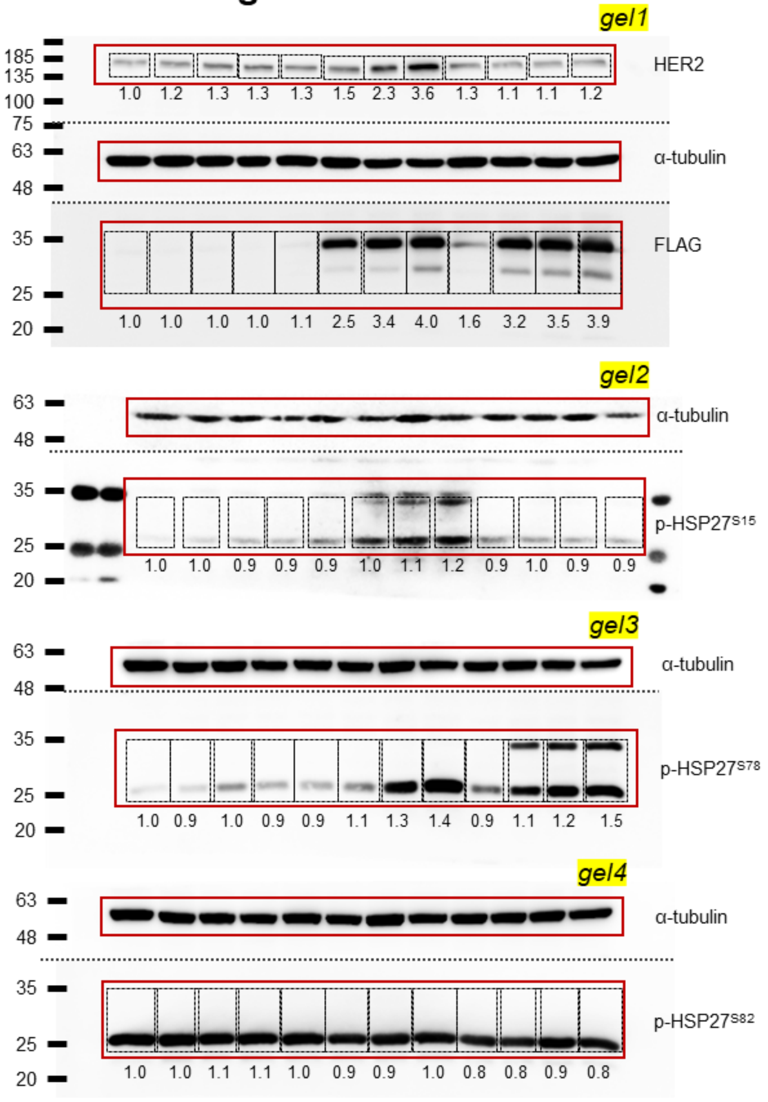

Related to Figure 4E

Black # : normalized by loading control
Purple # : normalized by total protein (phospho-protein/total protein)

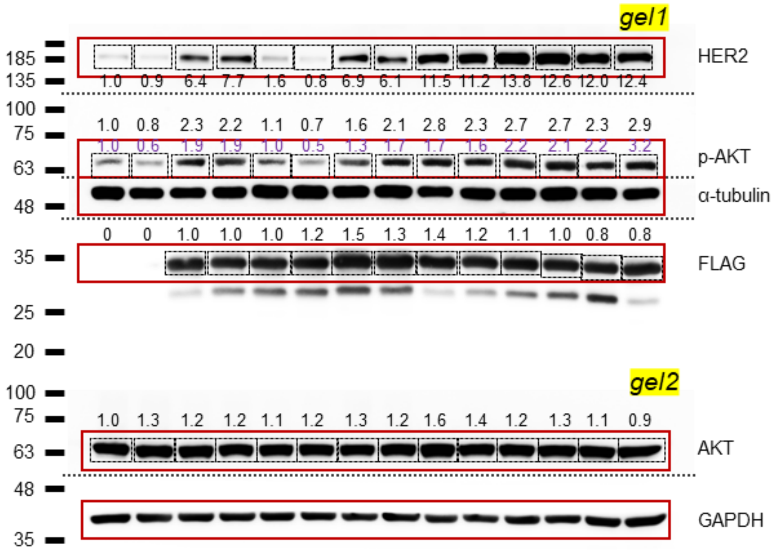

Related to Figure 5A

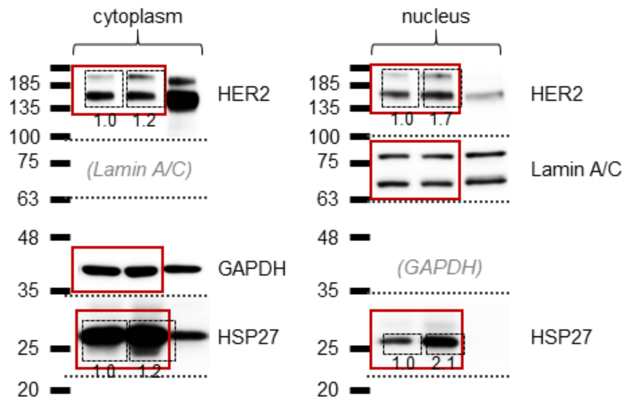

Related to Figure 5B

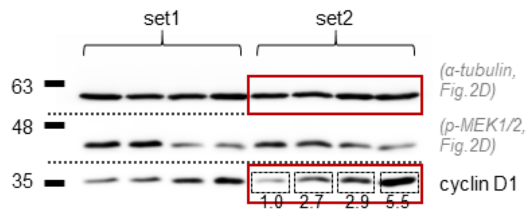

Related to Figure 5F

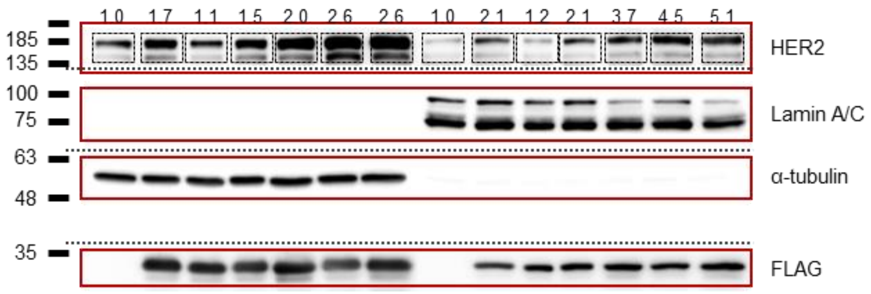

Related to Figure 5H

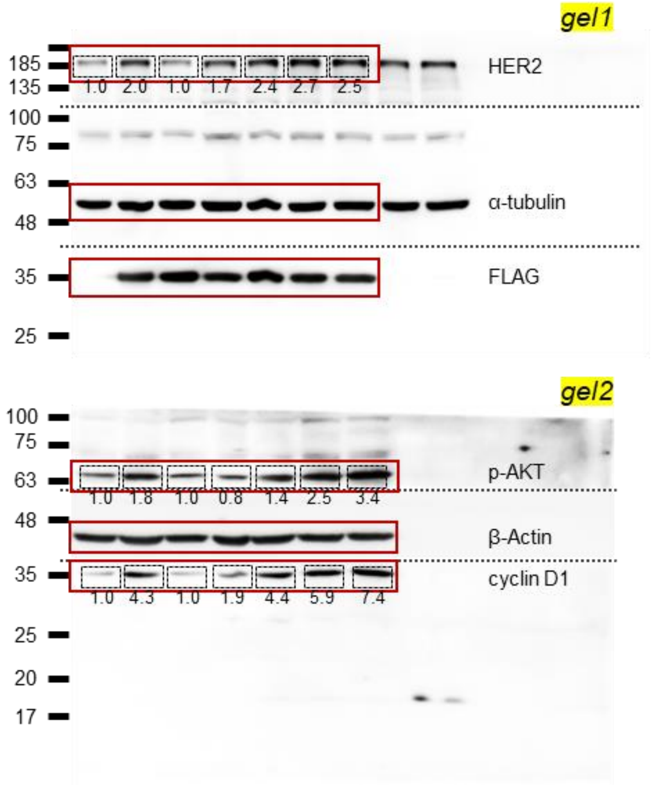

Related to Figure 6A

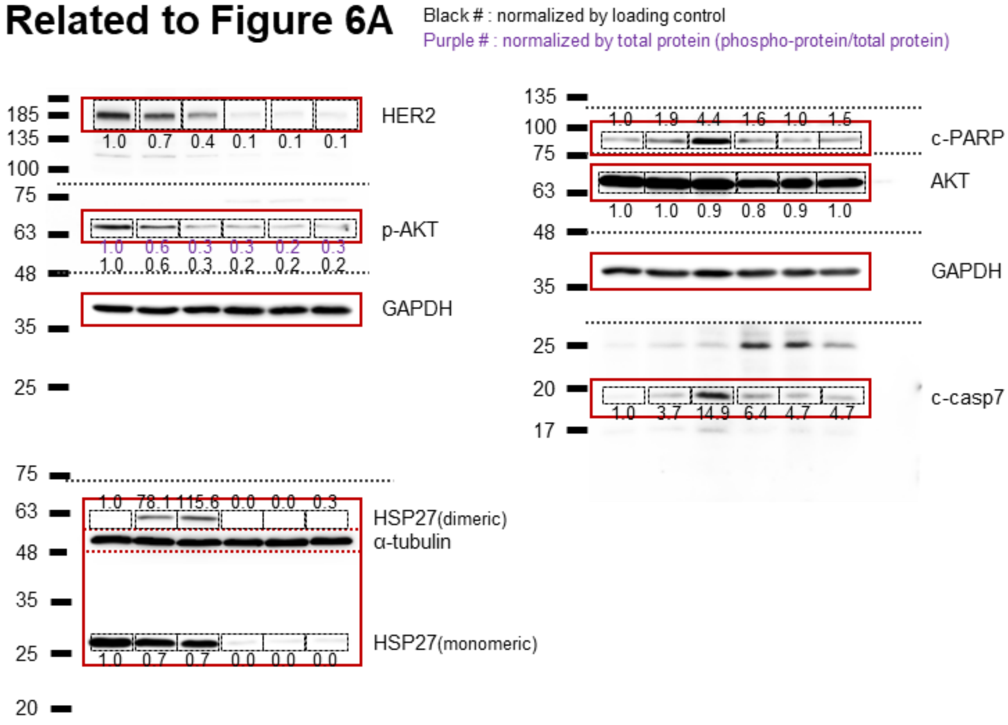

Related to Figure 6B

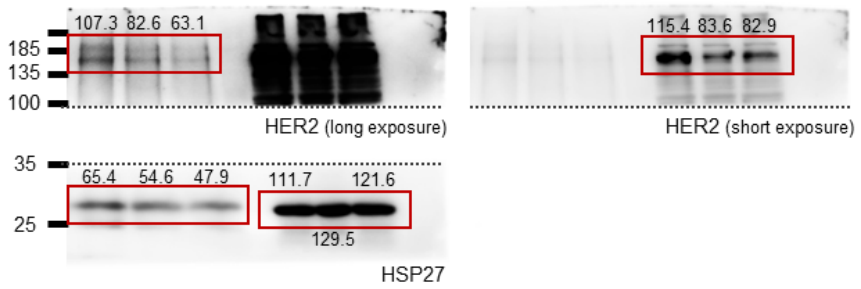

Related to Figure 6C

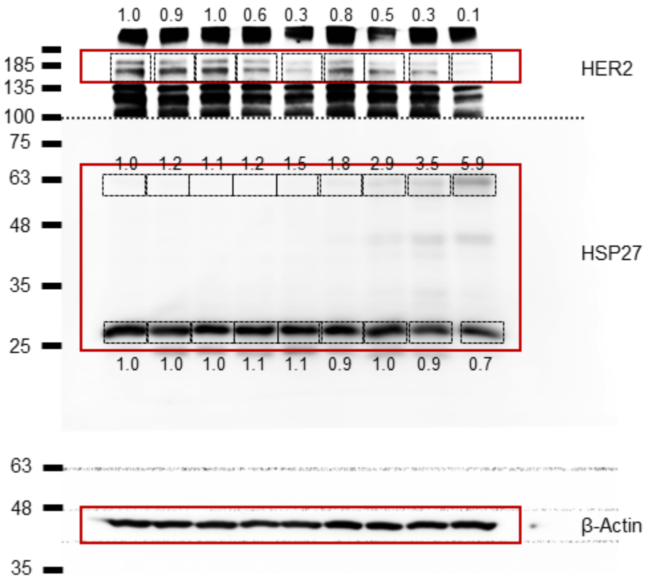

Related to Figure 6E

Black # : normalized by loading control
Purple # : normalized by total protein (phospho-protein/total protein)

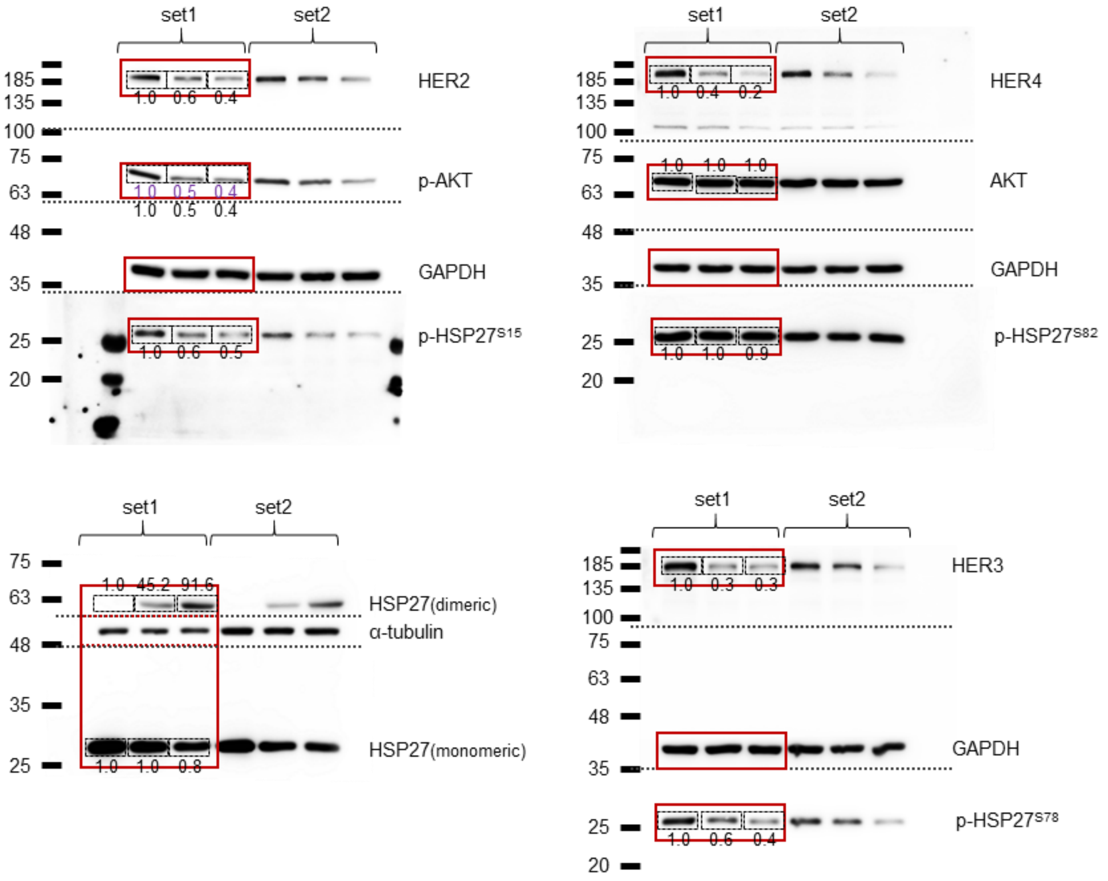

Related to Figure 6F

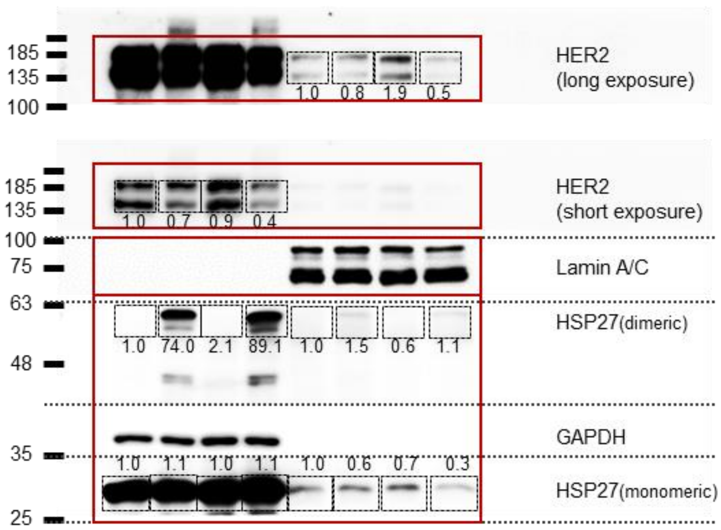

Related to Figure 6H, Figure S4D

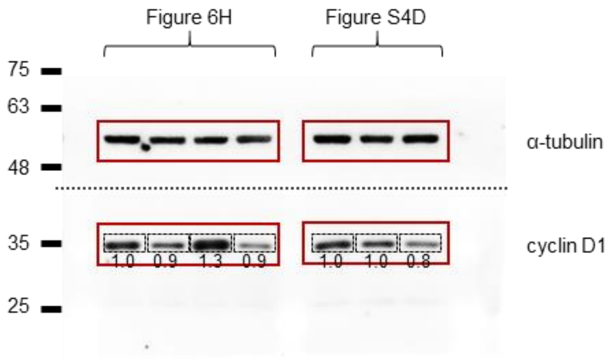

Related to Figure 7A

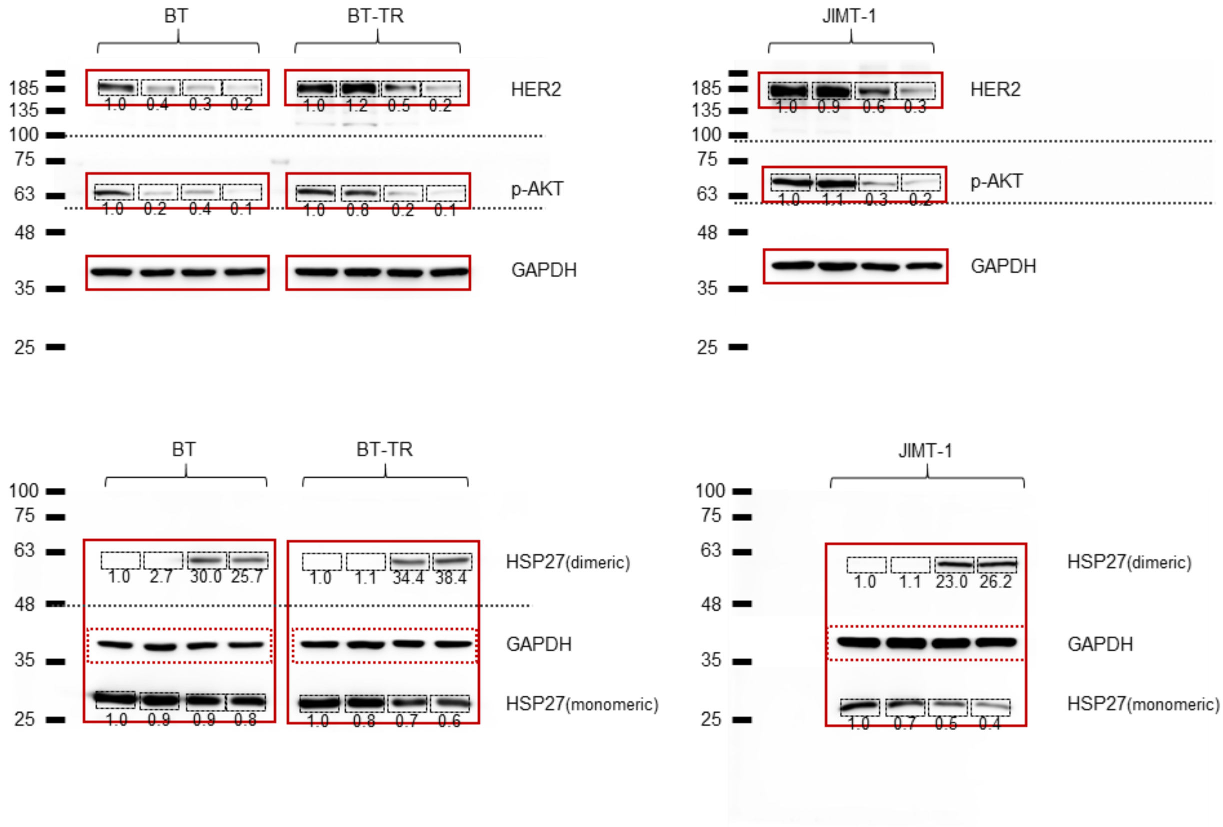

Related to Figure 7B

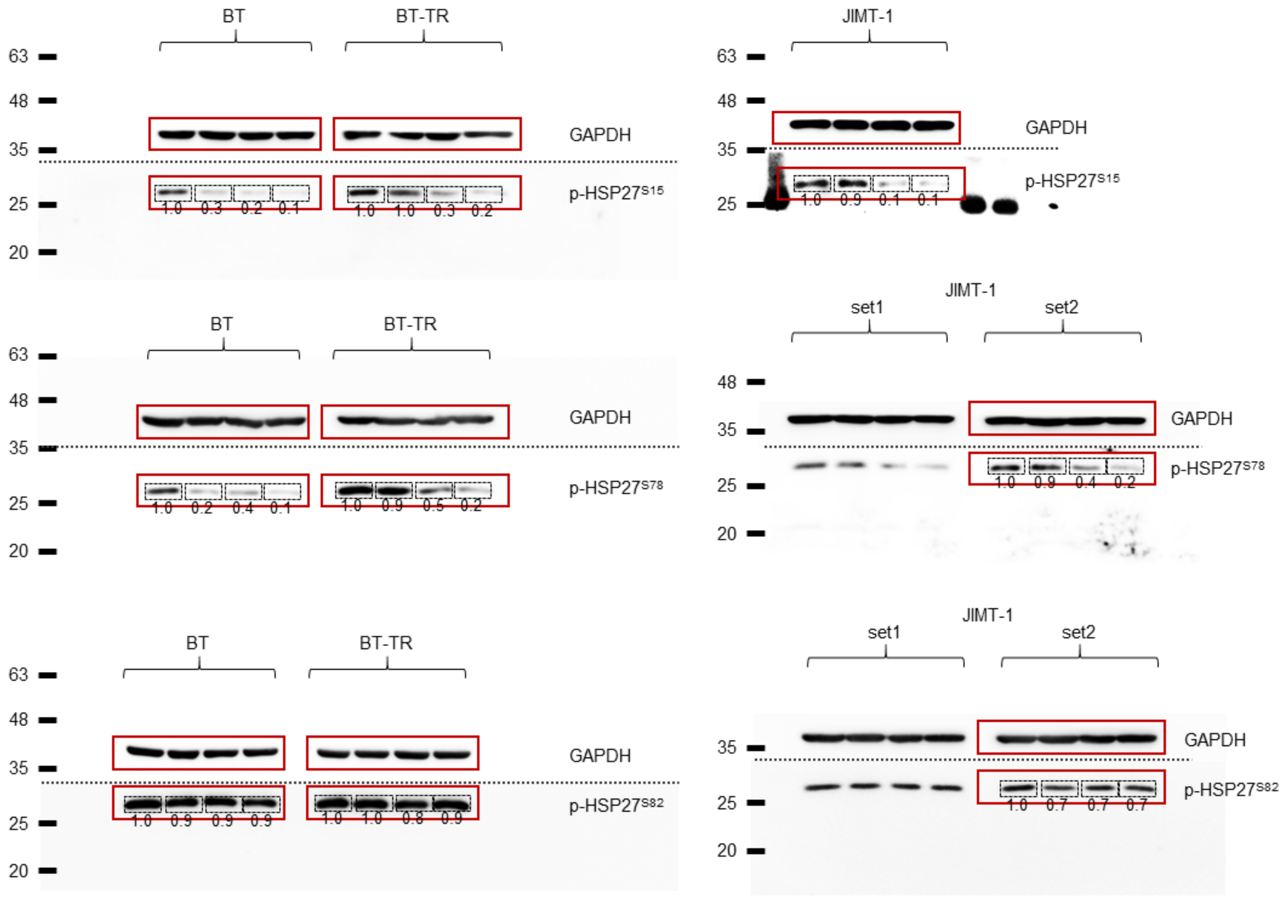

Related to Figure 7C

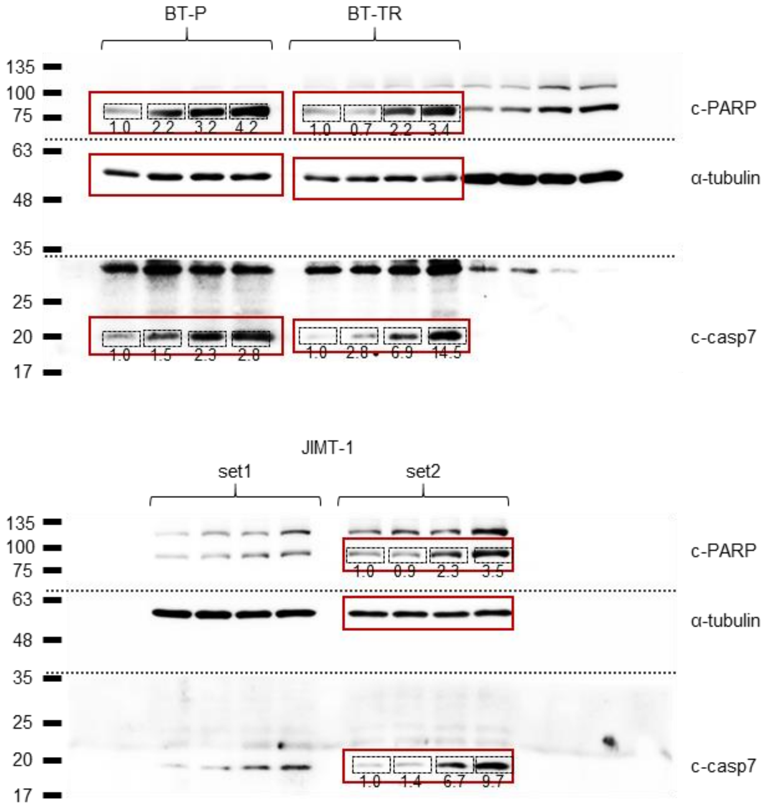

Related to Figure 8E

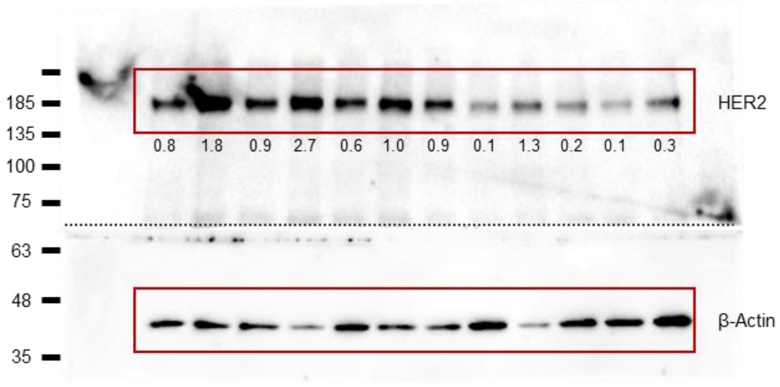

Related to Figure S3B

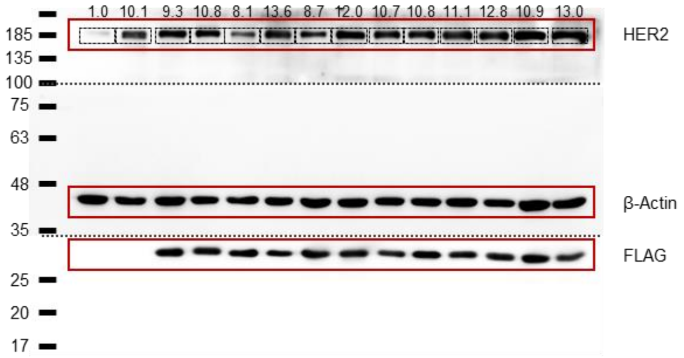

Related to Figure S4A

Black # : normalized by loading control
Purple # : normalized by total protein (phospho-protein/total protein)

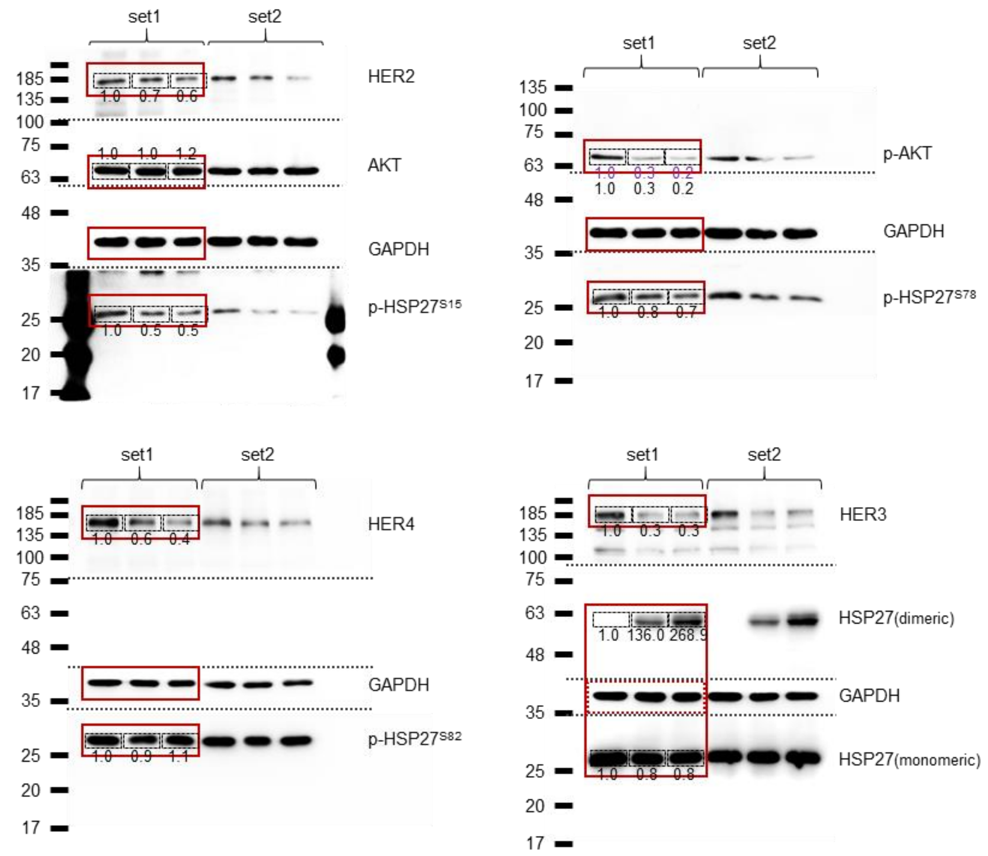

Related to Figure S4B

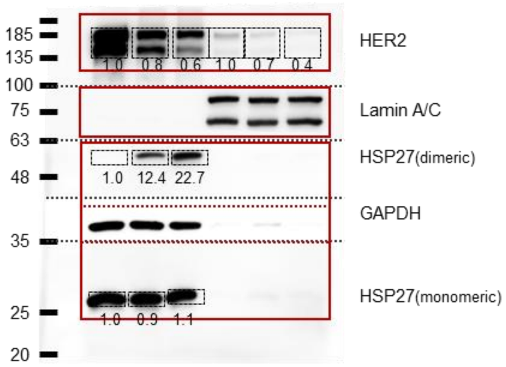

© 2019 by the authors. Submitted for possible open access publication under the terms and conditions of the Creative Commons Attribution (CC BY) license (http://creativecommons.org/licenses/by/4.0/).
